# Clinical utility of combinatorial pharmacogenomic testing in depression: A Canadian patient- and rater-blinded, randomized, controlled trial

**DOI:** 10.1038/s41398-022-01847-8

**Published:** 2022-03-14

**Authors:** Arun K. Tiwari, Clement C. Zai, C. Anthony Altar, Julie-Anne Tanner, Paige E. Davies, Paul Traxler, James Li, Elizabeth S. Cogan, Matthew T. Kucera, Ana Gugila, Nicole Braganza, Heather Emmerson, Gwyneth Zai, Daniel J. Müller, Robert Levitan, Stefan Kloiber, Zafiris J. Daskalakis, Benicio N. Frey, James M. Bowen, Jean-Eric Tarride, Richard Tytus, Ranjith Chandrasena, Nicholas Voudouris, Valerie H. Taylor, Raymond Tempier, Verinder Sharma, Akshya Vasudev, Peter Dzongowski, Lew Pliamm, Todd Greenspoon, Bryan M. Dechairo, James L. Kennedy

**Affiliations:** 1 Tanenbaum Centre for Pharmacogenetics, Neurogenetics Section, Molecular Brain Sciences Research Department, Campbell Family Mental Health Research Institute, Centre for Addiction and Mental Health, Toronto, ON Canada; 2 Department of Psychiatry, University of Toronto, Toronto, ON Canada; 3 Institute of Medical Science, University of Toronto, Toronto, ON Canada; 4 Laboratory Medicine and Pathobiology, University of Toronto, Toronto, ON Canada; 5 Splice Therapeutics, 20271 Goldenrod Lane, Lab 2071, Germantown, MD 20876 USA; 6 Myriad Neuroscience, Mason, OH USA; 7 Myriad Genetics, Salt Lake City, UT USA; 8 Campbell Family Mental Health Research Institute, Centre for Addiction and Mental Health, Toronto, ON Canada; 9 Department of Psychiatry, UC San Diego, San Diego, CA USA; 10 Mood Disorders Treatment and Research Centre and Women’s Health Concerns Clinic, St Joseph’s Healthcare Hamilton, Hamilton, ON Canada; 11 Department of Psychiatry and Behavioural Neurosciences, McMaster University, Hamilton, ON Canada; 12 Department of Health Research Methods, Evidence and Impact, Faculty of Health Sciences, McMaster University, Hamilton, Canada; 13 Program for Health System and Technology Evaluation, Ted Rogers Centre for Heart Research at Peter Munk Cardiac Centre, Toronto General Hospital Research Institute (TGHRI), Hamilton, Canada; 14 Toronto Health Economics and Technology Assessment (THETA) Collaborative, University Health Network (UHN), Hamilton, Canada; 15 Department of Health Research Methods, Evidence, and Impact (HEI), Faculty of Health Science, McMaster University, Hamilton, ON Canada; 16 Programs for Assessment of Technology in Health (PATH), The Research Institute of St. Joe’s Hamilton, St. Joseph’s Healthcare Hamilton, Hamilton, ON Canada; 17 Center for Health Economics and Policy Analysis (CHEPA), McMaster University, Hamilton, Canada; 18 Family Medicine, McMaster University, Hamilton, ON Canada; 19 Chatham-Kent Health Alliance, Chatham, ON Canada; 20 Thornhill Medical Center, Toronto, ON Canada; 21 Department of Psychiatry, University of Calgary, Hotchkiss Brain Institute, an Institute of the Cumming School of Medicine, Calgary, AB Canada; 22 Hôpital Montfort, Ottawa, ON Canada; 23 Department of Psychiatry, University of Ottawa, Ottawa, ON Canada; 24 Department of Psychiatry, Western University, London, ON Canada; 25 Department of Psychiatry, Geriatric Psychiatry and Neurosciences, Western University, London, ON Canada; 26 Integrative Psychiatry Lab, Parkwood Institute of Mental Health, London, ON Canada; 27 Milestone Research, London, ON Canada; 28 Canadian Phase Onward Inc., Polyclinic Family and Specialty Medicine Facility, Polyclinic Family Health Group, Toronto, ON Canada; 29 Hamilton Community Health Centre, Hamilton, ON Canada

**Keywords:** Personalized medicine, Pharmacogenomics

## Abstract

The pharmacological treatment of depression consists of stages of trial and error, with less than 40% of patients achieving remission during first medication trial. However, in a large, randomized-controlled trial (RCT) in the U.S. (“GUIDED”), significant improvements in response and remission rates were observed in patients who received treatment guided by combinatorial pharmacogenomic testing, compared to treatment-as-usual (TAU). Here we present results from the Canadian “GAPP-MDD” RCT. This 52-week, 3-arm, multi-center, participant- and rater-blinded RCT evaluated clinical outcomes among patients with depression whose treatment was guided by combinatorial pharmacogenomic testing compared to TAU. The primary outcome was symptom improvement (change in 17-item Hamilton Depression Rating Scale, HAM-D17) at week 8. Secondary outcomes included response (≥50% decrease in HAM-D17) and remission (HAM-D17 ≤ 7) at week 8. Numerically, patients in the guided-care arm had greater symptom improvement (27.6% versus 22.7%), response (30.3% versus 22.7%), and remission rates (15.7% versus 8.3%) compared to TAU, although these differences were not statistically significant. Given that the GAPP-MDD trial was ultimately underpowered to detect statistically significant differences in patient outcomes, it was assessed in parallel with the larger GUIDED RCT. We observed that relative improvements in response and remission rates were consistent between the GAPP-MDD (33.0% response, 89.0% remission) and GUIDED (31.0% response, 51.0% remission) trials. Together with GUIDED, the results from the GAPP-MDD trial indicate that combinatorial pharmacogenomic testing can be an effective tool to help guide depression treatment in the context of the Canadian healthcare setting (ClinicalTrials.gov NCT02466477).

## Introduction

The pharmacological treatment of depression consists of stages of trial-and-error prescribing, with less than 40% of patients achieving remission during their first medication trial and declining remission rates with each subsequent trial [1]. To help improve this state of affairs, organizations such as the Clinical Pharmacogenetics Implementation Consortium (CPIC), the Dutch Pharmacogenetics Working Group (KNMP), the United States (U.S.) Food and Drug Administration (FDA), and the International Society of Psychiatric Genetics (ISPG) provide recommendations and guidance on the use of pharmacogenetic information for several common psychotropic medications [2–6]. A growing body of evidence supports the use of pharmacogenomic testing to help guide the pharmacological treatment of depression and improve patients’ likelihood of achieving remission [7–9].

Combinatorial pharmacogenomic testing is distinct from other testing approaches, such as single- and multi-gene genetic testing, as it uses a weighted algorithmic assessment of multiple pharmacokinetic and pharmacodynamic genes to predict gene-drug interactions [10, 11]. The clinical utility of a combinatorial pharmacogenomic test has been assessed through both open-label and blinded clinical trials [10, 12–15]. This includes an evaluation of the clinical utility of combinatorial pharmacogenomic testing to guide the treatment of depression in the largest randomized controlled trial (RCT; *N* = 1167) of pharmacogenomic testing in psychiatry, performed in the U.S. from 2014 to 2017 [14]. Among patients with major depressive disorder (MDD) who failed at least one previous medication trial, the Genomics Used to Improve DEpression Decisions (GUIDED) trial demonstrated that combinatorial pharmacogenomic testing was associated with significant relative increases in both response (31%) and remission (51%) rates, compared to treatment-as-usual (TAU) [14]. Furthermore, meta-analyses have demonstrated that combinatorial pharmacogenomic testing is associated with a 49% relative increase in remission rates among patients with depression who have failed at least one previous medication trial when compared with patients who receive standard care [9].

The presence of universal healthcare in Canada, compared to the U.S., makes access to healthcare more equitable across socio-economic strata [16]. As the nature and cost of healthcare differs between the U.S. and Canada, several studies have evaluated the economic and clinical benefit of combinatorial pharmacogenomic testing in the Canadian system. The economic utility of combinatorial pharmacogenomic-guided treatment of depression in Canada has been demonstrated through studies of pharmacy claims and cost-effectiveness modeling [17, 18]. These studies report reduced healthcare costs in patients who receive treatment guided by combinatorial pharmacogenomic testing, compared to TAU. In terms of clinical utility, IMPACT (Individualized Medicine: Pharmacogenetics Assessment and Clinical Treatment), a large, single-arm, naturalistic study, demonstrated improvement in depression symptoms, response, and remission rates following combinatorial pharmacogenomic testing among Canadian patients with moderate to severe depression [15]. This single-arm study provides preliminary evidence of the clinical utility of combinatorial pharmacogenomic testing in Canada.

Although the existing evidence supports the effectiveness of combinatorial pharmacogenomic testing in a Canadian population, a direct evaluation in a patient- and rater-blinded RCT has not yet been reported. Here we present results from the Canadian Genomic Applications Partnership Program-Major Depressive Disorder (GAPP-MDD) RCT on the use of combinatorial pharmacogenomic testing to guide depression treatment. The GAPP-MDD trial evaluated clinical outcomes when treatment was guided by a combinatorial pharmacogenomic test (guided-care) compared to TAU among Canadian patients with MDD who had at least one previous failed medication trial.

## Materials and methods

### Trial design

The GAPP-MDD trial (ClinicalTrials.gov: NCT02466477) was a 52-week, three-arm, multi-center, patient- and rater-blinded, randomized, controlled trial evaluating clinical outcomes among patients whose treatment was guided by combinatorial pharmacogenomic testing compared to TAU. Patients were enrolled at screening between June 2015 and June 2018. Patient assessments were conducted at weeks 0 (baseline), 4, 8, 12, 24, 36, and 52. The analyses presented here focus on outcomes at Week 8. The 17-item Hamilton Depression Rating Scale (HAM-D17) was the primary assessment and was administered by blinded central rater. The self-rated 16-item Quick Inventory of Depression Symptomology (QIDS-SR_16_), a secondary assessment, was also administered at each visit. Additional description of the trial design has been reported in the Supplementary Materials.

The trial protocol was approved by Advarra research ethics board, Clinical Trials Ontario, Hamilton Integrated Research Ethics Board (HiREB), and IRB Schulman, and was performed in accordance with the principles of the Declaration of Helsinki. All patients provided informed consent after receiving a complete description of the study.

### Interventions: combinatorial pharmacogenomic testing

GeneSight® Psychotropic combinatorial pharmacogenomic testing was performed by Assurex Health Ltd. (Toronto, ON) on all patients enrolled in the GAPP-MDD trial, using previously described methods [19]. Standard testing (GEN) included select polymorphisms measured within 8 genes (*CYP1A2*, *CYP2B6*, *CYP2C9*, *CYP2C19*, *CYP2D6*, *CYP3A4*, *HTR2A*, and *SLC6A4*; U.S. patent no. 8,401,801 and U.S. patent no. 8,688,385). Seven additional polymorphisms within 6 genes shown to have genetic variation associated with antipsychotic-induced weight gain (*MC4R, CNR1, NPY, GCG, HCRTR2, NDUFS1*) [20–25] were measured for a subset of patients receiving “enhanced GeneSight” (EGEN) testing (U.S. patent no. 10,662,475). Genomic DNA was isolated from buccal samples, and the relevant genomic regions were amplified by polymerase chain reaction (PCR). Specific mutations for *CYP2B6* (A785G, G516T) and *SLC6A4* were detected by gel electrophoresis of PCR products. Analysis of *CNR1, CYP1A2*, *CYP2C19*, *CYP2C9*, *CYP3A4*, *GCG, HCRTR2, HTR2A, MC4R, NDUFS1*, and *NPY* was completed by using a custom xTAG® assay (Luminex Molecular Diagnostics). Analysis of *CYP2D6* was completed using xTAG® kits (Luminex Molecular Diagnostics). The following genetic variants may be detected in the assay: *CNR1 rs806378; CYP1A2 −3860G* > *A, −2467T* > *delT, −739T* > *G, −729C* > *T, −163C* > *A, 2116* *G* > *A, 2499* *A* > *T, 3497* *G* > *A, 3533* *G* > *A, 5090* *C* > *T, 5347* *C* > T; CYP2B6 *1, *4, *6, *9; CYP2C19 *1, *2, *3, *4, *6, *8, *17; CYP2C9 *1, *2, *3, *5, *6; CYP2D6 *1, *2, *2 A, *3, *4, *5, *6, *7, *8, *9, *10, *11, *12, *14, *15, *17, *41, gene duplication; CYP3A4 *1, *13, *15 A, *22; GCG rs13429709; HCRTR2 rs3134701, rs4142972; HTR2A −1438G > *A; MC4R rs489693; NDUFS1 rs6435326; NPY rs16147; SLC6A4 L, S* (Supplementary Table 1).

An algorithm weighed the combined influence of each individual genotype on patient response to each individual medication [10]. Based on this weighted and combined phenotype, 33 Health Canada-approved psychotropic medications (Supplementary Table 2) were categorized based on three levels of gene-drug interaction: “use as directed” (no gene-drug interactions), “use with caution” (moderate gene-drug interactions; i.e., medications may be effective with dose modification), and “use with increased caution and with more frequent monitoring” (severe gene-drug interactions that may significantly impact drug safety and/or efficacy).

### Randomization and blinding

Pharmacogenomic testing was performed for all patients between the screening and baseline visits. Patients were randomized 1:1:1 to one of three treatment arms, including two intervention arms and a TAU arm. The first intervention arm included patients for whom providers received the standard combinatorial pharmacogenomic test report to guide treatment (GEN arm). The second intervention arm included patients for whom providers received an enhanced combinatorial pharmacogenomic test report to guide treatment (EGEN arm; report included 6 additional genes shown to have genetic variation associated with antipsychotic-induced weight gain). Patients in the control arm (TAU) also received active treatment for their MDD; however, treatment did not include provision of the combinatorial pharmacogenomic test results and recommendations.

Both the patients and raters were blinded to the study arm. The treating clinician had knowledge of the intervention arm because the combinatorial pharmacogenomic report was available to them for patients in the GEN and EGEN arms to guide their treatment approach. In the TAU arm, patients and clinicians were blinded to the combinatorial pharmacogenomic test results until after completion of their week 36 visit.

### Participants

The detailed inclusion and exclusion criteria are described in the Supplementary Methods. Briefly, patients were included in the study if they were ≥18 years old, diagnosed with MDD (according to DSM-IV criteria, QIDS-C_16_ score ≥11 at screening, and QIDS-SR_16_ score ≥11 at screening and baseline), and had inadequate response to at least one psychotropic medication included on the combinatorial pharmacogenomic report within the current depressive episode. Patients were excluded if they had significant suicidal risk, severe co-occurring psychiatric or cognitive disorders, and/or unstable or significant medical conditions. Patients were recruited from both psychiatric care and primary care settings.

### Outcomes

The following outcomes were pre-specified in the Statistical Analysis Plan, available on clinicaltrials.gov as of April 23, 2019 (NCT02466477). The primary outcome was symptom improvement, defined as the mean percent change in HAM-D17 score from baseline to week 8, compared between the GEN and TAU arms in the per-protocol (PP) cohort. Symptom improvement was also compared between GEN and TAU in the intent-to-treat (ITT) cohort. Additionally, the mean absolute change in HAM-D17 score from baseline to week 8 was compared between GEN and TAU arms in both cohorts.

Symptom improvement from baseline to week 8 and response and remission rates at week 8 were compared between the EGEN and GEN arms. If no statistically significant difference was observed between the EGEN and GEN arms for any of these outcomes, it was pre-specified that the two arms would be combined into the single “guided-care” arm for all of the subsequent analyses.

The following secondary patient outcomes were compared between the study arms at week 8 in both the PP and ITT cohorts: (1) Response at week 8, defined as ≥ 50% decrease in HAM-D17 from baseline, and (2) Remission at week 8, defined as having a score of ≤ 7 for HAM-D17. Symptom improvement, response, and remission, defined using the HAM-D17 scale, were also evaluated at week 24. The proportion of patients taking only congruent medications at baseline and week 4 and 8 follow-up were assessed in the study arms in both the PP and ITT cohorts.

The number needed to treat (NNT) for response and remission was defined as the number of subjects required to receive combinatorial pharmacogenomic testing in order for one more subject in the GEN or EGEN treatment arms to achieve the clinical outcome (i.e., response or remission) above that observed in the TAU group.

### Statistical analysis

#### Analysis cohorts

Patient outcomes were assessed in the following pre-specified cohorts: PP and ITT. As specified in the study protocol, QIDS-C_16_ was not conducted at baseline and therefore not included as a requirement for either cohort. The ITT cohort included patients who met eligibility criteria, with the exception of the baseline QIDS-SR_16_ < 11 requirement; patients with a QIDS-SR_16_ < 11 were included in analyses as randomization occurred before baseline. As the intention of the trial was to assess the clinical utility of the combinatorial pharmacogenomic test in patients with moderate or worse depression, the PP cohort excluded patients who did not meet this criterion. Therefore, the PP cohort included the subset of patients who met eligibility criteria and additionally excluded patients who had baseline scores <14 on the HAM-D17 to exclude patients with mild depression. Patients were also excluded if they had clinically relevant protocol violations, as described in CONSORT Diagrams (Supplementary Figs 1, 2), or if their clinician did not view the electronic combinatorial pharmacogenomic report prior to the baseline visit (GEN and EGEN arms only, for the latter).

The final ITT and PP cohorts included 371 patients, and 276 patients, respectively. Patient recruitment was terminated prior to achieving the target enrollment sample size (*N* = 570), which was based on effect sizes of an early open-label study of pharmacogenomic testing, as described in the Supplementary Methods. The early termination of this trial was based on analyses of results from the large GUIDED trial, which was similar in design to the GAPP-MDD trial, and had the same primary outcome. Primary results for the GUIDED trial were reported in May 2018, when the GAPP-MDD trial was still actively enrolling. Using the effect size observed in the GUIDED trial, power was reassessed, and the target sample size for the GAPP-MDD study was found to be powered at less than 25% probability to detect a statistically significant difference between arms. To achieve 90% power, approximately *N* = 4000 patients would be required, which was not feasible for this trial.

#### Medication congruence with combinatorial pharmacogenomic testing

Medication congruence was based on whether a medication was subject to gene-drug interactions as determined by the combinatorial pharmacogenomic test results for a given patient [11]. Gene-drug interaction refers to genetic variation that is predicted to impact the pharmacokinetics or pharmacodynamics of a medication. Incongruent medications were classified as those that were subject to significant gene-drug interactions for the given patient. Patients were classified as taking congruent medications if none of their prescribed medications were subject to significant gene-drug interactions (i.e., none of their medications were classified in the “use with increased caution and with more frequent monitoring” bin on the pharmacogenomic report).

#### Data analysis

All analyses were performed according to a pre-specified statistical analysis plan, available on clinicaltrials.gov as of April 23, 2019 (NCT02466477). Patient demographics and clinical characteristics were assessed using descriptive statistics for the PP and ITT cohorts. A 3-sample test for the equality of proportions was used to assess differences between the 3 treatment arms after randomization for categorical measures and one-way ANOVA was used for continuous measures. A 2-sample test for the equality of proportions was used to compare the number of patients who previously failed three or more medications between treatment arms. Analyses were performed for patients who completed the study through week 8. The severity of depression was categorized according to HAM-D17 scores: 0–7, normal; 8–13, mild depression; 14–18, moderate depression; 19–22, severe depression; ≥23, very severe depression.

A Mixed Model for Repeated Measures (MMRM) including treatment group, week, baseline HAM-D17 score, treatment group-by-week interaction, and baseline HAM-D17-by-week interaction was used for both change and percentage change from baseline in HAM-D17. A generalized linear mixed model including treatment group, week, baseline HAM-D17 score, treatment group-by-week interaction, and baseline HAM-D17-by-week interaction was used for response and remission in HAM-D17. A Chi-Square test was used for medication congruence analyses. Two-sided *P* values ≤ 0.05 were considered statistically significant. Analyses were performed using SAS software (Version 9.4) and JMP 15 (SAS Institute).

#### Evaluation of GAPP-MDD and GUIDED

As the design of the Canadian GAPP-MDD trial resembled that of the large U.S. GUIDED trial, [14] these studies were assessed in parallel. In order to evaluate the similarities in patient outcomes between the two trials, endpoints from each study (i.e., outcomes at week 8) are presented here in a side-by-side table when similar data were available (Supplementary Table 3). As described previously, part of the rationale for the current study was to evaluate the clinical utility of combinatorial pharmacogenomic testing to guide depression treatment in the context of the Canadian healthcare system. Therefore, including the U.S. GUIDED results provides context to the Canadian GAPP-MDD results.

#### Meta-analysis

A meta-analysis was performed to compare the findings from the GAPP-MDD trial with those from previous similarly designed RCTs. Clinical outcomes were compared between baseline and week 8 for the GAPP-MDD and GUIDED [14] trials, and week 10 for the Winner et al. trial [12]. Symptom improvement from baseline was measured by the percentage change in HAM-D17 from baseline. Mean percentage of symptom improvement at week 8 or week 10 comparing the combinatorial pharmacogenomic-guided care arm to TAU (treatment as usual) and standard error of the mean from each study were used for the meta-analysis. For response and remission, the odds ratio (OR) comparing the combinatorial pharmacogenomic-guided care arm to TAU at week 8 or week 10 with standard error for each study was used for analyses. The pooled mean effect of symptom improvement and pooled odds ratios of response and remission for the three studies were calculated using a fixed effects model. The fixed effects model was used as the study designs were the same and the studies all estimate the same treatment effect. Heterogeneity in effect sizes across studies were tested using the Q-statistic and its magnitude was measured with the I2 statistic. Meta-analyses were run using the meta package in R version 4.0.2. The NNT for each study was calculated using the following equation: 1/AAR, where AAR (Absolute Attributable Risk) was the absolute value of (CER – EER); CER (Control Event Rate) was the event rate (i.e., number of patients achieving response or remission) in the TAU arm and EER (Experimental Event Rate) was the event rate in the combinatorial pharmacogenomic test-guided arm. The NNT for all studies was calculated using the combined CER and EER across the three studies.

## Results

### Cohort description

Study enrollment and follow-up for patients in the PP and ITT cohorts are presented in CONSORT diagrams (Supplementary Figs 1, 2). A total of 570 patients were planned for enrollment, with 190 in each of the TAU, GEN, EGEN arms in accordance with the protocol. After early termination of enrollment due to subsequent determination of lack of power, the final ITT cohort consisted of 371 patients who completed the baseline visit (*N* = 118 TAU, *N* = 125 GEN, *N* = 128 EGEN; Supplementary Fig. 1). In the PP cohort, an additional 95 patients were excluded after randomization as they had a baseline HAMD-17 score <14 or had other protocol violations. Therefore, the final per-protocol cohort consisted of 276 patients (*N* = 93 TAU, *N* = 90 GEN, *N* = 93 EGEN; Supplementary Fig. 2).

Although the primary analyses focus on the PP cohort, the results from the ITT analyses are also shown here for reference. The baseline demographic characteristics of the PP cohort are presented in Table 1. The same information for the ITT cohort can be found in Supplementary Table 4. In the PP cohort, the majority of patients were female (64.5%), 18–64 years of age (93.9%; mean age 41 years), and self-reported as “Caucasian” (84.1%). The mean HAM-D17 score was 21.4, with 30.4% of patients classified as having moderate, 27.5% severe, and 42% very severe depression. Generalized anxiety disorder was the most common psychiatric comorbidity (43.1%). The mean number of lifetime previously failed psychiatric medications was 3.6. The rate of three or more failed medications in the TAU arm was 46.7% (42/90) compared to 60% in GEN + EGEN arm (108/180; *p* = 0.051). A total of 196 patients in the PP cohort had HAM-D17 scores available at the week 8 endpoint (*N* = 69 TAU, *N* = 65 GEN, *N* = 62 EGEN), and 149 patients had HAM-D17 scores available through the blinded week 24 endpoint (*N* = 55 TAU, *N* = 48 GEN, *N* = 46 EGEN).

**Table 1:** Demographic characteristics in the GAPP-MDD clinical trial at baseline by treatment in the per-protocol cohort.

Demographics	Treatment			Total (*N* = 276)	*p*-value
	TAU (*N* = 93)	GEN (*N* = 90)	E-GEN (*N* = 93)		
Age group, *n* (%)					
18 to 34	35 (37.6)	42 (46.7)	32 (34.4)	109 (39.5)	0.21
35 to 49	27 (29.0)	21 (23.3)	32 (34.4)	80 (29.0)	0.26
50 to 64	25 (26.9)	20 (22.2)	25 (26.9)	70 (25.4)	0.71
65 and over	6 (6.5)	7 (7.8)	4 (4.3)	17 (6.2)	0.61
Age					
Mean (SD)	42.3 (14.2)	40.3 (15.3)	40.7 (12.9)	41.1 (14.1)	0.62
Min, Max	20.0, 78.0	19.0, 76.0	18.0, 69.0	18.0, 78.0	–
Gender, *n* (%)					
Female	59 (63.4)	59 (65.6)	60 (64.5)	178 (64.5)	0.96
Male	34 (36.6)	31 (34.4)	33 (35.5)	98 (35.5)	0.96
Ethnicity, *n* (%)^a^					
Asian	7 (7.5)	10 (11.1)	7 (7.5)	24 (8.7)	0.61
Black	1 (1.1)	3 (3.3)	4 (4.3)	8 (2.9)	0.40
Caucasian	83 (89.2)	72 (80.0)	77 (82.8)	232 (84.1)	0.21
Latin American	2 (2.2)	2 (2.2)	1 (1.1)	5 (1.8)	0.81
Other	0	3 (3.3)	4 (4.3)	7 (2.5)	0.15
Depression category, *n* (%)					
Moderate (HAM-D17 14–18)	28 (30.1)	26 (28.9)	30 (32.3)	84 (30.4)	0.88
Severe (HAM-D17 19–22)	25 (26.9)	24 (26.7)	27 (29.0)	76 (27.5)	0.92
Very severe (HAM-D17 > 22)	40 (43.0)	40 (44.4)	36 (38.7)	116 (42.0)	0.71
Psychiatric comorbidities, *n* (%)					
Generalized anxiety disorder	35 (37.6)	45 (50.0)	38 (40.9)	119 (43.1)	0.23
Panic disorder	8 (8.6)	15 (16.7)	15 (16.1)	38 (13.8)	0.21
Post-traumatic stress disorder	7 (7.5)	7 (7.8)	8 (8.6)	22 (8.0)	0.96
HAM-D17					
Mean (SD)	21.4 (4.5)	21.3 (4.7)	21.5 (4.8)	21.4 (4.7)	0.99
Min, Max	14.0, 36.0	14.0, 36.0	14.0, 33.0	14.0, 36.0	–
Number of failed psych meds, *n*					
1	28	14	20	62	0.0598
2	20	23	15	58	0.29
3	12	8	14	34	0.44
4	9	17	8	34	0.07
5	8	7	10	25	0.77
6+	13	20	24	57	0.12
Missing^b^	3	1	2	6	0.62
Mean (SD)	3.0 (2.2)	3.7 (2.2)	4.0 (3.1)	3.6 (2.6)	0.0388
Min, Max	1.0, 9.0	1.0, 8.0	1.0, 21.0	1.0, 21.0	–

^a^Expanded ethnicity groupings are presented in Supplementary Tables 10–12.

^b^Patient who reported failing ≥1 prior medication trial, but did not report specific number of prior failed trials.

### Clinical outcomes

There was no significant difference in the primary endpoint, percent decrease in HAM-D17 score, between the GEN and TAU arms at week 8 in the PP cohort (GEN 24.4 vs TAU 22.6, *p* = 0.738), nor was there a difference in the mean absolute decrease in HAM-D17 score between GEN and TAU (GEN 5.2 vs TAU 5.1, *p* = 0.901; Table 2). Similar results were found in the ITT cohort (Table 2).

**Table 2:** Week 8 symptom improvement in the GEN and TAU treatment arms, according to HAM-D17.

	Symptom improvement					
	Mean percentage change			Mean change		
	Estimate,% (SE)^a^	Difference	*p*-value	Estimate (SE)^b^	Difference	*p*-value
Per Protocol						
GEN (*N* = 65)	24.41 (3.85)	1.82	0.738	5.23 (0.74)	0.13	0.901
TAU (*N* = 69)	22.60 (3.80)			5.10 (0.73)		
Intent-to-Treat						
GEN (*N* = 106)	22.42 (3.42)	4.61	0.350	4.70 (0.57)	0.672	0.414
TAU (*N* = 97)	17.80 (3.55)			4.03 (0.59)		

^a^Mean percent change in HAM-D17 score from baseline to week 8.

^b^Mean absolute change in HAM-D17 score from baseline to week 8.

When comparing the GEN and EGEN arms at week 8 in the PP cohort, there was no significant difference in HAM-D17 endpoints (symptom improvement, *p* = 0.244; response, *p* = 0.332; remission, *p* = 0.834; Supplementary Table 5). Furthermore, due to the similarity between the combinatorial pharmacogenomic algorithms used in the GEN and EGEN arms, there was no difference at week 8 in medication binning on the pharmacogenomic report according to the two algorithms (i.e., medications categorized as “use as directed”, “use with caution”, or “use with increased caution and with more frequent monitoring”). At week 24, only one patient was taking a medication that changed bins in the GEN and EGEN algorithms, but this did not impact their congruence categorization. Based on the above, and as prespecified in the Statistical Analysis Plan, the GEN and EGEN arms were combined for the remainder of the study and referred to as the guided-care arm.

**Fig. 1:**
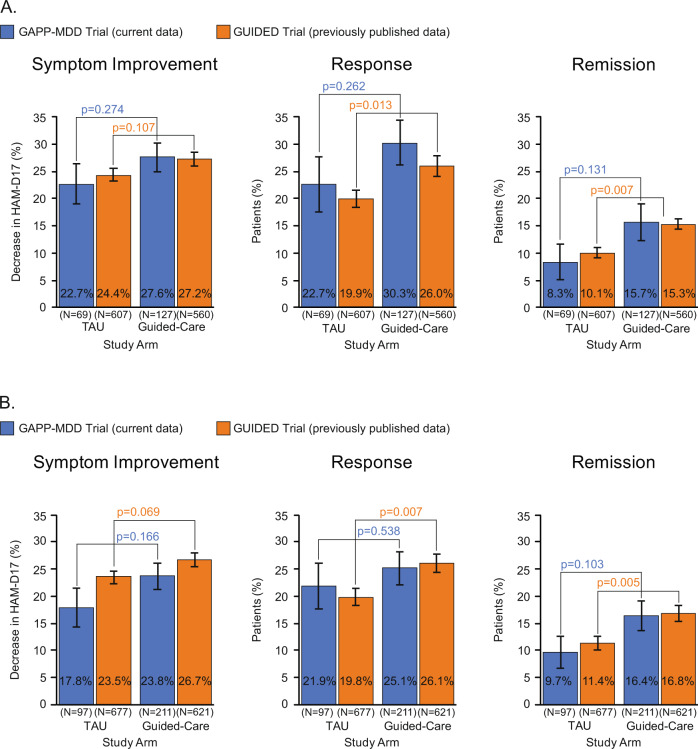
HAM-D17 clinical outcomes at 8 weeks by treatment arm in the GAPP-MDD clinical trial and the previous GUIDED trial [14]. **A** Per-protocol (PP) cohort. GAPP-MDD trial: Guided care arm *N* = 127, TAU arm *N* = 69; GUIDED trial: Guided care arm *N* = 560, TAU arm *N* = 607. **B** Intent-to-treat (ITT) cohort. GAPP-MDD trial: Guided care arm *N* = 211, TAU arm *N* = 97); GUIDED trial: Guided care arm *N* = 621, TAU arm *N* = 677.

The key outcomes comparing the guided-care and TAU arms at week 8 in the GAPP-MDD study are provided in Fig. 1 and Supplementary Table 3. To help interpret these results and provide additional clinical context, the corresponding data from the prior GUIDED study are also presented. As shown, in the PP cohort of the current GAPP-MDD study, compared to TAU, patients in the guided-care arm had greater HAM-D17-measured symptom improvement (27.6% versus 22.7%; *p* = 0.274), higher response rate (30.3% versus 22.7%; *p* = 0.262), and higher remission rate (15.7% versus 8.3%; *p* = 0.131), although comparisons did not reach statistical significance. Nonetheless, these statistically non-significant values in the GAPP-MDD study are in the direction of improvement, with relative improvements of 22% in symptoms, 33% in response rates, and 89% in remission rates among patients in the guided-arm, compared to TAU. Findings in the ITT cohort at week 8 were similar.

The durability of clinical outcomes was also compared between the guided-care and TAU arms at week 24 (Supplementary Tables 6, 7). In the PP cohort, patients in the guided-care arm experienced an improvement in HAM-D17-measured symptom improvement, response, and remission rates compared to patients in the TAU arm, although no differences reached statistical significance. In the ITT cohort, patients in the guided-care arm continued to experience greater HAM-D17-measured symptom improvement, response, and remission through week 24 compared to patients in the TAU arm, although these improvements did not reach statistical significance.

### Decision impact regarding medication congruence

**Fig. 2:**
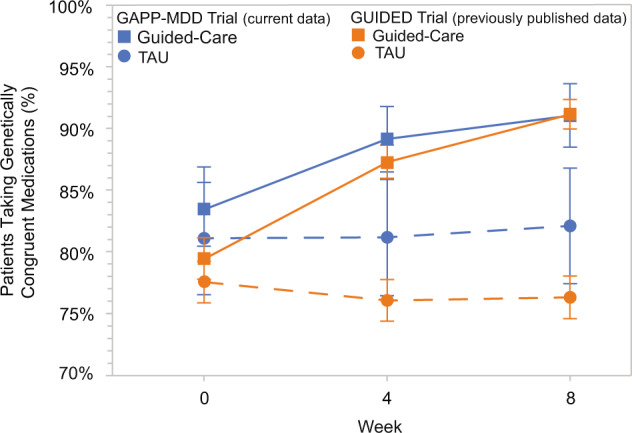
Medication congruency by week in the Per-Protocol cohort of the GAPP-MDD clinical trial and the previous GUIDED trial [14]. The proportion of patients taking genetically congruent medications from the GAPP-MDD and GUIDED trials are shown in blue and orange, respectively. Squares represent Guided-Care treatment arms, and circles represent TAU treatment arms.

The proportion of patients taking congruent medications at baseline and week 8 was compared between the guided-care and TAU arms in the PP cohort (Fig. 2; Supplementary Table 8). Congruent prescribing increased over the course of the study in the guided-care arm but not in the TAU arm. In the guided-care arm, 83.4% of patients were taking genetically congruent medications at baseline, which increased to 91.1% at week 8. Conversely, in the TAU arm, 81.1% of patients were taking genetically congruent medications at baseline, and 82.1% were taking congruent medications at week 8 (*p* = 0.070 for the guided-care arm compared to TAU at week 8). Similar results were observed within the ITT cohort (Fig. 2; Supplementary Table 8).

### Clinical outcomes in the GAPP-MDD and GUIDED RCTs

As the design of the GAPP-MDD trial resembled that of the U.S. GUIDED trial, clinical outcomes and congruency were presented for the two studies in parallel. The improvement in clinical outcomes observed in the PP cohort of the GAPP-MDD trial was similar to what was observed in the GUIDED trial (Supplementary Table 9). In fact, improvement in HAM-D17-measured symptom improvement, response, and remission at week 8 in the guided-care arm compared to TAU was numerically greater in the GAPP-MDD trial, compared to GUIDED (Fig. 1; Supplementary Table 3). In addition, the increase in medication congruence within the guided-care arms was consistent between studies. Findings within the ITT cohort for GAPP-MDD and GUIDED were also similar (Fig. 1; Supplementary Table 3).

### Meta-analysis

**Fig. 3:**
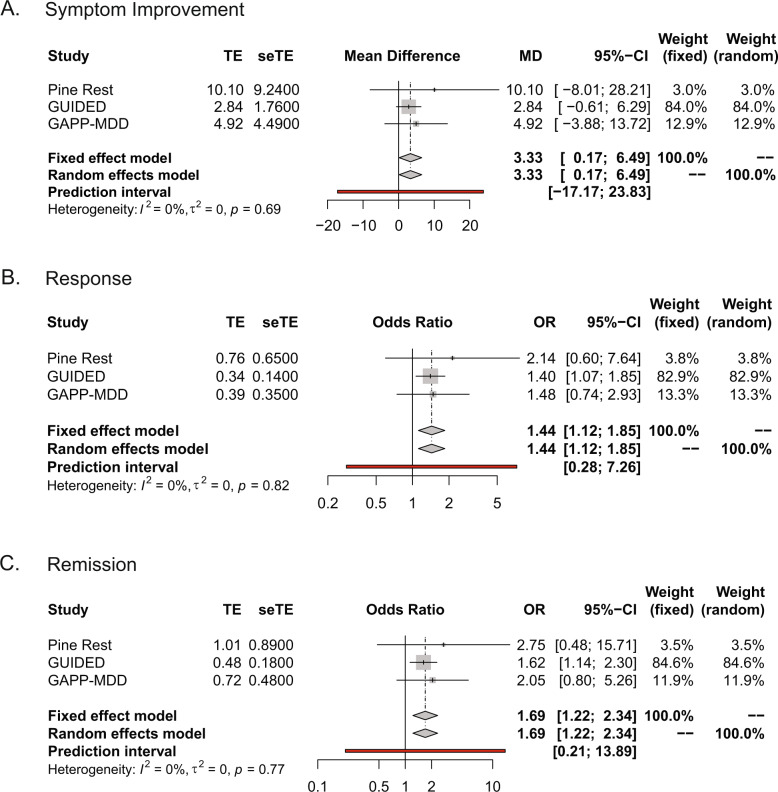
Meta-analysis of randomized-controlled trials evaluating combinatorial pharmacogenomic testing. **A** Meta-analysis of symptom improvement. **B** Meta-analysis of response. **C** Meta-analysis of remission.

The primary end point of the three studies was symptom improvement, measured as percent change from baseline HAMD-17 score to week 8 (for GAPP-MDD and GUIDED trials) or 10 (for Winner et al. 2013). The effect size of symptom improvement was consistent across the three studies (*Q* = 0.74; *p* = 0.69; I^2^ = 0). The fixed effects model suggests that symptom improvement was significantly better when combinatorial pharmacogenomic test results were available to the physician, with HAM-D17 scores decreasing an additional 3.33% (95% CI: 0.17–6.49; *p* = 0.039) from baseline to week 8 or 10 for guided-care versus TAU (Fig. 3A).

Overall response based on HAM-D17 was also evaluated for the three studies, defined as at least a 50% reduction in HAM-D17 score from baseline to week 8 or 10. The effect size of response was consistent across the three studies (Q = 0.41; *p* = 0.82; I^2^ = 0). The pooled odds ratio from the fixed effects model suggests the guided-care arm had a 44% relative improvement in response rate compared with the TAU arm (OR = 1.44, 95% CI: 1.12–1.85; *p* = 0.004; Fig. 3B). The NNT (i.e., number of patients needed in the GEN and EGEN arms for one more patient to achieve clinical outcomes) for the response was 13.16 for GAPP-MDD, 16.39 for GUIDED, and 6.58 for Pine Rest. The combined NNT for response across the three studies was 14.47.

All three studies also evaluated remission, defined as HAM-D17 ≤ 7 at week 8 or 10. The effect size of remission was consistent across the three studies (Q = 0.524; *p* = 0.77; I^2^ = 0). The pooled odds ratio from the fixed effects model suggests that the guided-care arm achieved a 69% relative improvement in remission compared to TAU (OR = 1.69, 95% CI: 1.22–2.34; *p* = 0.001; Fig. 3C). The NNT for remission was 13.51 for GAPP-MDD, 19.23 for GUIDED, and 8.55 for Pine Rest. The NNT for remission across all three studies combined was 17.62.

## Discussion

In both the U.S. and Canada, pharmacotherapy is a first-line treatment option for patients suffering from MDD [26, 27]. However, approved medications can differ across countries, along with healthcare pathways and costs associated with treatment. Therefore, it is important to evaluate the clinical utility of combinatorial pharmacogenomic testing and confirm the U.S. findings in other populations, including Canadian patients with MDD. The GAPP-MDD trial presented clinical outcomes among MDD patients in Canada whose treatment was guided by combinatorial pharmacogenomic testing compared to TAU. Although this trial was underpowered to detect statistically significant differences, numeric improvements in depression symptoms, response rate, and remission rates were observed among patients in the guided-care arm (i.e., who received treatment guided by combinatorial pharmacogenomic testing), compared to TAU. Combinatorial pharmacogenomic-guided treatment also resulted in positive treatment decision impact for prescribing clinicians, where the proportion of patients taking congruent medications increased to over 90% in the guided-care arm, with no change in the TAU arm.

As the intention of the GAPP-MDD trial was to evaluate the utility of the combinatorial pharmacogenomic test in a Canadian population, this trial was assessed in conjunction with a trial conducted in a U.S. population (GUIDED trial). In the GAPP-MDD trial, relative improvements observed in depression symptoms, response, and remission rates in the guided-care arm were consistent with those reported in GUIDED. The GUIDED trial was a larger RCT (*N* = 1167) and was, therefore, better powered to achieve statistical significance. The results from the GAPP-MDD trial were consistent with significant improvements in response and remissions rates from the GUIDED trial, even though the GAPP-MDD trial, itself, was statistically underpowered. Notably, of the outcomes assessed, we observed the most improvement in remission rates in both studies (51% in GUIDED, statistically significant; 89% in GAPP-MDD, not statistically significant). This is particularly relevant, as remission, the resolution of depression symptoms, is the most challenging endpoint to achieve when treating MDD, and the Canadian Network for Mood and Anxiety Treatments (CANMAT) identifies symptom remission as the primary target of the acute treatment of MDD [28]. The findings from the GAPP-MDD trial, although not statistically significant, are broadly consistent with other studies in the field. Given that trial-and-error is the most common strategy for antidepressant prescribing, even small gains in response can be clinically meaningful. The meta-analysis contributes to the growing body of evidence on the clinical usefulness of combinatorial pharmacogenomics testing [10, 12–15, 29, 30].

Changes in clinicians’ prescribing of genetically congruent medications in GAPP-MDD paralleled that of the GUIDED trial. Both studies reported a similar increase in clinicians prescribing congruent medications for patients in the guided-care arm, but not TAU arm. This demonstrates consistency in the decision impact of combinatorial pharmacogenomic testing when clinicians have the test available to guide treatment decisions. The high baseline medication congruence in both studies may have resulted from the trial and error involved in selecting an average of 3.6 prior medications per patient; this may have further resulted in more patients on congruent medications. Still, the number of patients in the guided-care arm who were prescribed non-congruent (i.e., genetically discordant) medications decreased by 50% during the study, whereas no decrease occurred in patients in the TAU arm.

A notable difference between the GAPP-MDD and GUIDED RCTs is the ethnicity composition of the patient cohorts. In the PP cohort of both studies, the majority of patients reported “Caucasian” as their ethnicity (GAPP-MDD 84.1%, GUIDED 73.5%). Additionally, the second most common self-reported ethnicity was different between the two studies (“Asian” in the GAPP-MDD cohort, 8.7%; “Black” in the GUIDED cohort, 14.5%). There was also a considerable difference in the proportion of patients who identified as “Latin American” (GAPP-MDD 1.8%, GUIDED 7.9%) between studies. These distributions of patient ethnicities are largely consistent with those observed on a population level within the respective country in which each study was run [31, 32]. Despite differences in self-reported ethnicity between the GAPP-MDD and GUIDED trials, we observed similar improvements in patient outcomes in both cohorts.

One important component of both the GAPP-MDD and GUIDED RCTs is the assessment of outcomes in both the PP and ITT cohorts. In the GAPP-MDD trial, the demographic characteristics of the PP and ITT cohorts were comparable, with the exception that patients with no or mild depression were included in the ITT cohort. Additionally, all patients in the PP cohort had moderate, severe, or very severe depression, whereas 13% of the patients in the ITT cohort had no or mild depression, as determined by baseline HAM-D17 scores of 0–7 and 8–13, respectively. Despite some demographic differences between each of the two cohorts, results were similar, with improved clinical outcomes in the guided-care arm, compared to TAU.

There were several limitations to the current study. The statistical power and design of the GAPP-MDD was determined using effect size estimates for symptom improvement (mean percent change in HAM-D17 score from baseline to week 8) from an earlier open-label clinical trial of combinatorial pharmacogenomic testin﻿g [13]. However, as the GAPP-MDD trial was in progress, it became apparent upon release of data from the much larger GUIDED RCT, that the current trial was underpowered based on the GUIDED RCT effect size for symptom improvement. However, we include the GUIDED trial results in this report to provide additional clinical context and relevance. Additional limitations of the GAPP-MDD trial include the following: the majority of the cohort were “Caucasian” (as determined by self-report); lack of assessment of adherence; the impact of polypharmacy on outcomes was not evaluated; and the sample size in the PP and the ITT populations differed due to exclusion of patients with no or mild depression from the PP analyses (although results were similar in both cohorts). In addition, positive expectations are known to influence treatment response. Since the clinicians were not blinded to study arm, positive expectations might have subtle influence on the observations made in this study. A potential strength of the GAPP-MDD study is that this patient population received care under the universal Canadian healthcare system; therefore, socioeconomic biases related to accessibility and affordability of care may have been reduced (although this may have varied by specific study site).

In summary, we observed similar improvement in clinical outcomes following combinatorial pharmacogenomic testing in a Canadian population of patients with MDD who failed to respond to at least one previous medication trial as was seen in the large U.S. GUIDED trial. The similar effect sizes in response and remission between the two studies indicate that the differences observed in the GAPP-MDD trial may represent true differences between arms that did not reach statistical significance due to cohort size. Therefore, results from the GAPP-MDD trial, conducted in the context of the Canadian universal healthcare setting, together with evidence from the GUIDED trial, indicate that combinatorial pharmacogenomic testing is an additional tool available to clinicians that provides clinically useful information to help guide the treatment of depression.

## Supplementary information

Supplementary Materials
