# Correction: Clinical utility of combinatorial pharmacogenomic testing in depression: A Canadian patient- and rater-blinded, randomized, controlled trial

**DOI:** 10.1038/s41398-022-01963-5

**Published:** 2022-05-30

**Authors:** Arun K. Tiwari, Clement C. Zai, C. Anthony Altar, Julie-Anne Tanner, Paige E. Davies, Paul Traxler, James Li, Elizabeth S. Cogan, Matthew T. Kucera, Ana Gugila, Nicole Braganza, Heather Emmerson, Gwyneth Zai, Daniel J. Müller, Robert Levitan, Stefan Kloiber, Zafiris J. Daskalakis, Benicio N. Frey, James M. Bowen, Jean-Eric Tarride, Richard Tytus, Ranjith Chandrasena, Nicholas Voudouris, Valerie H. Taylor, Raymond Tempier, Verinder Sharma, Akshya Vasudev, Peter Dzongowski, Lew Pliamm, Todd Greenspoon, Bryan M. Dechairo, James L. Kennedy

**Affiliations:** 1 Tanenbaum Centre for Pharmacogenetics, Neurogenetics Section, Molecular Brain Sciences Research Department, Campbell Family Mental Health Research Institute, Centre for Addiction and Mental Health, Toronto, ON Canada; 2 Department of Psychiatry, University of Toronto, Toronto, ON Canada; 3 Institute of Medical Science, University of Toronto, Toronto, ON Canada; 4 Laboratory Medicine and Pathobiology, University of Toronto, Toronto, ON Canada; 5 Splice Therapeutics, 20271 Goldenrod Lane, Lab 2071, Germantown, MD 20876 USA; 6 Myriad Neuroscience, Mason, OH USA; 7 Myriad Genetics, Salt Lake City, UT USA; 8 Campbell Family Mental Health Research Institute, Centre for Addiction and Mental Health, Toronto, ON Canada; 9 Department of Psychiatry, UC San Diego, San Diego, CA USA; 10 Mood Disorders Treatment and Research Centre and Women’s Health Concerns Clinic, St Joseph’s Healthcare Hamilton, Hamilton, ON Canada; 11 Department of Psychiatry and Behavioural Neurosciences, McMaster University, Hamilton, ON Canada; 12 Department of Health Research Methods, Evidence and Impact, Faculty of Health Sciences, McMaster University, Hamilton, Canada; 13 Program for Health System and Technology Evaluation, Ted Rogers Centre for Heart Research at Peter Munk Cardiac Centre, Toronto General Hospital Research Institute (TGHRI), Hamilton, Canada; 14 Toronto Health Economics and Technology Assessment (THETA) Collaborative, University Health Network (UHN), Hamilton, Canada; 15 Department of Health Research Methods, Evidence, and Impact (HEI), Faculty of Health Science, McMaster University, Hamilton, ON Canada; 16 Programs for Assessment of Technology in Health (PATH), The Research Institute of St. Joe’s Hamilton, St. Joseph’s Healthcare Hamilton, Hamilton, ON Canada; 17 Center for Health Economics and Policy Analysis (CHEPA), McMaster University, Hamilton, Canada; 18 Family Medicine, McMaster University, Hamilton, ON Canada; 19 Chatham-Kent Health Alliance, Chatham, ON Canada; 20 Thornhill Medical Center, Toronto, ON Canada; 21 Department of Psychiatry, University of Calgary, Hotchkiss Brain Institute, an Institute of the Cumming School of Medicine, Calgary, AB Canada; 22 Hôpital Montfort, Ottawa, ON Canada; 23 Department of Psychiatry, University of Ottawa, Ottawa, ON Canada; 24 Department of Psychiatry, Western University, London, ON Canada; 25 Department of Psychiatry, Geriatric Psychiatry and Neurosciences, Western University, London, ON Canada; 26 Integrative Psychiatry Lab, Parkwood Institute of Mental Health, London, ON Canada; 27 Milestone Research, London, ON Canada; 28 Canadian Phase Onward Inc., Polyclinic Family and Specialty Medicine Facility, Polyclinic Family Health Group, Toronto, ON Canada; 29 Hamilton Community Health Centre, Hamilton, ON Canada

**Keywords:** Personalized medicine, Pharmacogenomics

Correction to: *Translational Psychiatry* 10.1038/s41398-022-01847-8, published online 14 March 2022

The original version of this article unfortunately contained mistakes. To ensure the accuracy of their article the authors would like to make the following corrections: Correction #1 Page 5, paragraph 1, line 5: Text currently reads “136”. This is incorrect and the authors are requesting to change it to “149”. Correction #2 Page 7, paragraph 4, line 17: Text currently reads “mild and moderately depressed patients”. This is incorrect and the authors are requesting to change it to: “patients with no or mild depression”. Correction #3 Supplementary Materials, Supplementary Table 2: The following Antipsychotic medications are incorrectly listed under Antidepressants: Olanzapine, Paliperidone, Perphenazine, Quetiapine, Risperidone, Thiothixene, Ziprasidone. The authors are requesting to update Supplementary Table 2 to include the above Antipsychotic medications in the Antipsychotic medication section of the table. The authors apologize for the errors. The original article has been corrected accordingly.

