# Supplementary material for: Clinical utility of combinatorial pharmacogenomic testing in depression: A Canadian patient- and rater-blinded, randomized, controlled trial
Source: Transl Psychiatry. 2022 Mar 14;12:101. doi: 10.1038/s41398-022-01847-8 (PMC8921325; doi:10.1038/s41398-022-01847-8)
Supplement: Supplementary file 1 — Supplementary Materials [file 41398_2022_1847_MOESM1_ESM.docx]

**Supplementary Methods**

*Trial Design*

The GAPP-MDD trial (ClinicalTrials.gov: NCT02466477) was a 52-week, three-arm, multi-center, patient- and rater-blinded, randomized, controlled trial evaluating clinical outcomes among patients with major depressive disorder (MDD) whose treatment was guided by combinatorial pharmacogenomic testing compared to TAU.

Patient assessments were conducted at week 0 (baseline), 4, 8, 12, 24, 36, and 52. The 17-item Hamilton Depression Rating Scale (HAM-D17) was the primary assessment and was administered by blinded central rater. The following secondary assessments were also administered: Patient Health Questionnaire (PHQ-9; patient reported) and the self-rated 16-item Quick Inventory of Depression Symptomology (QIDS-SR_16_).

The trial protocol was approved by Advarra research ethics board, Clinical Trials Ontario, Hamilton Integrated Research Ethics Board (HiREB), and IRB Schulman, and was performed in accordance with the principles of the Declaration of Helsinki. All patients provided informed consent after receiving a complete description of the study.

*Sample size calculation*

The original sample size calculations from the protocol were as follows. Assuming an effect size of 0.30 in HAM-D17 score favoring the treatment group (based on an early study of pharmacogenomic testing in major depression^1^), intra class coefficient between clusters of 20%, statistical power of 90%, an alpha level of 0.05, and an expected 16.7% rate of premature discontinuation (based on ongoing studies and experience with the intervention) by week 8 (primary endpoint), a total of 570 participants (i.e., 190 per treatment arm) are required to detect the same effect in this study. Sample size calculations were determined using SAS (SAS Institute, Inc., Version 9.3, Cary, NC).

*Study Site Selection and Subject Population*

Patients were recruited from 8 academic and community hospital outpatient clinics, family health teams, and primary health care clinics in Ontario between June 2015 and June 2018. Patients were eligible for inclusion in this study if they met the following criteria: 18 years of age or older at the time of screening; had total screening score of ≥11 on the QIDS-C16 and total screening and baseline scores of ≥11 on QIDS-SR16 rating scales; suffer from a Major Depressive Episode meeting DSM-IV-TR criteria; have had an inadequate response within current episode to at least one psychotropic medication (listed in Supplementary Table 2); able to understand requirements of study and provide informed consent to participate in study; and agree to abide by study protocol and complete all aspects of study.

Patients meeting any of the following exclusion criteria were not eligible to participate: suicidal risk; bipolar I or II disorder; current Axis I diagnosis of delirium; dementia; amnestic and other cognitive disorder; schizophrenia or other psychotic disorder; hallucinations, delusions, or any psychotic symptomatology within the current or prior depressive episodes; currently in an inpatient facility; history of hypothyroidism (unless on stable dose of thyroid medication and asymptomatic or euthyroid for 6 months); significant substance use disorder according to DSM-IV-TR criteria; or significant unstable medical condition or other significant medical conditions such as life-threatening disease, hepatic insufficiency, liver transplant, cirrhosis of the liver, malignancy, chemotherapy within 1 year prior to screening, or need for therapies that may obstruct results of treatments and/or of the study; participation in another clinical trial within 30 days of screening visit; anticipated inability to attend scheduled study visits; unreliable or uncooperative with protocol as judged by Investigator; history of prior pharmacogenomics testing; any change in psychotropic medication between screening and baseline; currently receiving or scheduled to receive ECT, DBS, or TMS during course of study; pregnant or lactating; or history of gastric bypass surgery.

*Randomization and Blinding*

Upon completion of screening procedures, subjects meeting study entry criteria were randomized 1:1:1 to one of three treatment arms, including two intervention arms and a TAU arm. The first intervention arm included patients for whom providers received the standard combinatorial pharmacogenomic test report to guide treatment (GEN arm). The second intervention arm included patients for whom providers received an enhanced combinatorial pharmacogenomic test report to guide treatment (EGEN arm; report included 6 additional genes shown to have genetic variation associated with antipsychotic-induced weight gain). Randomization occurred before the baseline visit to ensure PGx report could be generated and reviewed by the treating clinician prior to the baseline visit. Randomization was performed by a computer-generated randomization schedule stratified by site using permuted blocks of random sizes. An unblinded programmer at PATH or HMD Clinical uploaded patient randomization into the electronic data capture (EDC) system. A confirmatory randomization code was generated, and an email was sent to authorized personnel at Assurex Health Ltd.

Before baseline, the treating clinician received the results of the GEN test for patients allocated to GEN arm and the results of the E-GEN test for patients allocated to the E-GEN arm, and may have decided to change the patient’s treatment based on the results of the test. Therefore, the treating clinician was not blinded to treatment allocation. In the TAU arm, patients and clinicians were blinded to the combinatorial pharmacogenomic test results until after completion of their week 36 visit. Authorized personnel at Assurex Health Ltd. were not blinded to treatment arm in order to release the appropriate report to the treatment clinician and to provide follow-up support to the treating clinician on report interpretation. However, both the treating clinician and Assurex Health Ltd. were blinded to the trial data. Both the patients and raters (site and centralized raters) were blinded to the study arm.

*Intervention: Combinatorial Pharmacogenomics*

*GeneSight® Psychotropic (GEN)*

Patients randomized to the GEN group had buccal swabs of their cheeks taken by the clinician and mailed to the Assurex Health Ltd. (ARx) pharmacogenomics lab in a secure envelope labeled with a unique identifier to ensure accuracy and validity. Once received, the pharmacogenomic laboratory analyzed the sample and determined the patient’s genotypes and corresponding phenotypes for each of the genes tested on the GEN panel. These phenotypes provide a clear picture of how functional each gene is with respect to drug metabolism (for CYP450 genes) and/or drug response (for neurotransmitter transporter and receptor genes).

After laboratory testing was completed and phenotypes were assigned, GEN technology categorized the green, yellow, or red status of each drug and the footnote(s) that accompanied each drug. This was accomplished by integrating the genetic data with the pharmacology for each medication on the GEN panel (**Supplementary Table 2**), and incorporating data gleaned from Health Canada-approved labels and published literature.

Within three days of sample receipt at the laboratory, results were returned via the GeneSight clinician portal to the treating clinicians in the form of an interpretive report that categorized medications into three independent bins titled “Use as Directed”, the Green Bin, “Use With Caution”, the Yellow Bin, and “Use with Increased Caution And With More Frequent Monitoring”, the Red Bin. Medications in the Green Bin are least likely to be affected by genetic polymorphisms identified in the tested individual. Medications in the Yellow Bin are at risk for gene-drug interactions that may necessitate alternative dosing or medication selection. Medications in the Red Bin are at higher risk for a gene-drug interaction that may necessitate alternative dosing or medication selection. Guided by these results, the treating physician may have decided to modify a participant’s psychotropic medication selection, dose, dose regimen and/or number of medications.

*Enhanced-GeneSight Psychotropic (EGEN)*

All procedures for patients randomized to the E-GEN arm were the same as for patients randomized to the GEN arm except that for E-GEN, the pharmacogenomic laboratory also analyzed each patient’s genotypes and corresponding phenotypes for the 7 CAMH markers for AIWG. The treating clinicians received an interpretive report that reflects all of the E-GEN genes. Guided by the results from E-GEN, the treating physician may have decided to modify a participant’s psychotropic medication, in terms of type of medication, dose, dose regimen and/or number of medications.

Treatment as Usual (TAU)

Patients randomized to the TAU arm also had their DNA collected and a pharmacogenomics-based interpretive report was generated using GEN testing. However, this report was not shared with the treating clinicians until up to 28 days prior to the completion at 12 months of the study at which point the report was provided to the participant by the treating clinician. Therefore, patients in this arm received clinical treatment as usual, without the use or knowledge of genotyping results by their treating clinicians.

*Outcomes*

All assessments were administered by the site, with the exception of HAM-D17 (the primary outcome measure), which was conducted via telephone by 1-2 trained central raters not involved in the clinical care of the patient and blinded to the treatment allocation. The raters were trained, and inter-rater reliability was determined through rater scoring of the HAM-D17 while viewing a DVD-recording of a standardized patient interview. The HAM-D17 was administered at baseline and at the end of weeks 4, 8, 12, 24, 36, and 52.

The 17-item Hamilton Depression Rating Scale (HAM-D17), which remains a gold standard used in trial of anti-depressants, was the primary efficacy endpoint for this study and assesses mood, feelings of guilt, insomnia, agitation or retardation, anxiety, weight loss, somatic symptoms and suicidal ideation. Secondary efficacy variables included the self-rated 16-item Quick Inventory of Depression Symptomology (QIDS-SR16) rating scale; and 9-item Patient Health Questionnaire (PHQ-9, patient reported). Exploratory, safety, and tolerability variables included the Generalized Anxiety Disorder 7-item (GAD-7, patient reported); Clinical Global Impression of Severity (CGI-S) (collected by the treating clinician); Clinical Global Impression of Improvement (CGI-I, collected by the treating clinician); Clinical Global Impression Efficacy Index (CGI-EI, collected by the treating clinician); Short Form Health Survey (SF-36, patient reported); Udvalg for Kliniske Undersogeler Side Effect Rating Scale (UKU, patient reported); Frequency, Intensity, and Burden of Side Effects Ratings (FIBSER, patient reported); weight and hip-to-waist ratio; EuroQol (EQ-5D-5L, patient reported); and the Pharmacogenetics in Psychiatry Follow-Up Questionnaire (PIP-FQ, clinician rated). Safety assessments included potential clinical events that were spontaneously reported by patients or observed during the course of study assessments.

*Data Collection and Management*

Data was collected using a validated electronic data capture (EDC) solution. Electronic case report forms (eCRFs) were utilized for recording data from each subject meeting the eligibility criteria and being randomized into the study. Electronic access to the CRF was available to all Investigator sites. All study staff responsible for entering data into the eCRF system were trained prior to the start-up of the study. A personal log-in was provided for all responsible personnel to allow for an audit trail relating to the study data to be maintained.

All evaluations performed were entered into the eCRF by a member of the site staff delegated responsibility for this specific task by the Principal Investigator of the clinical site. It was the responsibility of the Investigator to ensure that the eCRFs were properly completed. The data in the eCRFs was consistent with the relevant source documents. The Investigator signed the designated signature fields of the eCRF to confirm that the information on each screen was accurate and complete. All data was stored in an unidentifiable form treated with strict confidentiality in accordance with applicable data-protection regulations.

Captured data was monitored electronically and source data verification (SDV) took place at the site where all information was verified against the individual patient records. Any inconsistencies were presented as queries; either as automatically generated queries if raised by the logical data checks of the eCRF system, or by manually generated queries if raised by the data validation checks or the SDV performed by the data manager (DM) or the CRA, respectively. Queries were resolved by a trained member of the site staff.

**Supplementary Figure 1. CONSORT Flow Diagram for the Intent-to-treat cohort.***


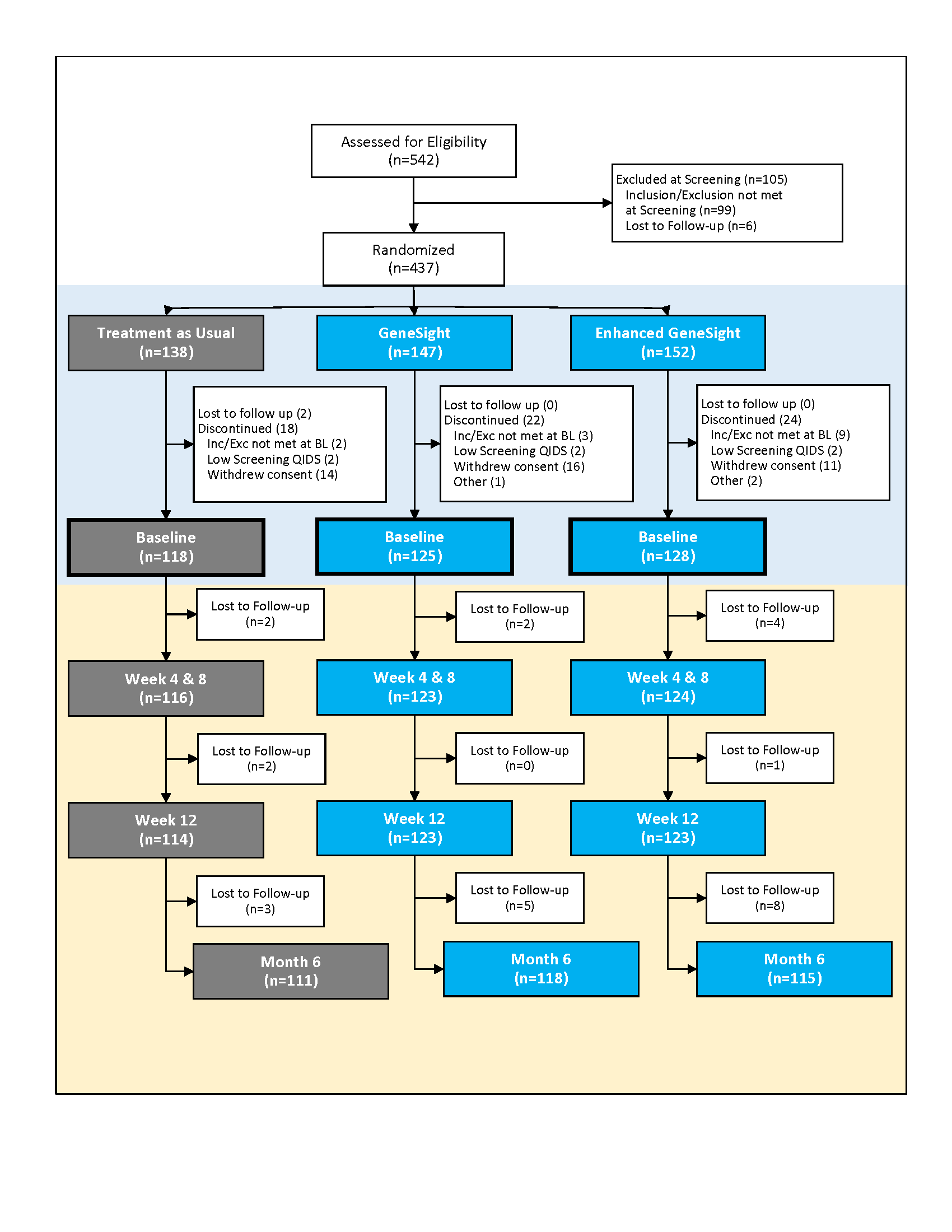
* The number of patients reported in the CONSORT diagram represents the total number of patients in the study at each time point, regardless of missing data points. Therefore, the number of patients in the analyses for Weeks 4, 8, 12, and Month 6 may be lower than those reported in the CONSORT diagram.

**Supplementary Figure 2. CONSORT Flow Diagram for the Per-Protocol cohort.***


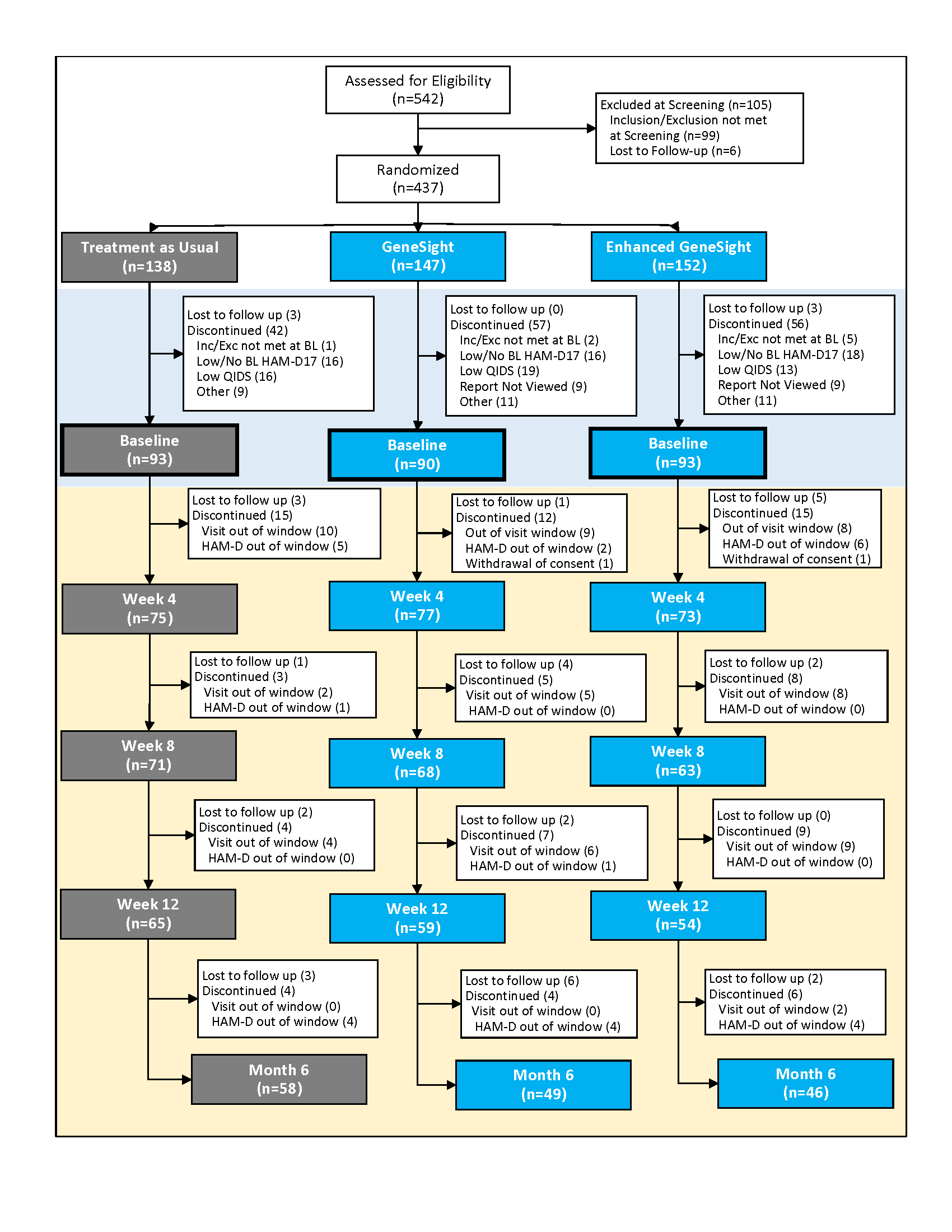


* The number of patients reported in the CONSORT diagram represents the total number of patients in the study at each time point, regardless of missing data points. Therefore, the number of patients in the analyses for Weeks 4, 8, 12, and Month 6 may be lower than those reported in the CONSORT diagram.

**Supplementary Table 1: The dbSNP rs# and PharmVar nomenclature for the alleles analyzed for the pharmacogenomic report.**

| **Gene Sight** | **Gene** | **rs number** | **PharmVar^a^ nomenclature** |
| --- | --- | --- | --- |
| rs806378C>T | CNR1 | rs806378 | n/a |
| -3860G>A | CYP1A2 | rs2069514 | CYP1A2*1C |
| -2467T>delT | CYP1A2 | rs35694136 | CYP1A2*1D |
| -739T>G | CYP1A2 | rs2069526 | CYP1A2*1E |
| -729C>T | CYP1A2 | rs12720461 | CYP1A2*1K |
| -163C>A | CYP1A2 | rs762551 | CYP1A2*1F |
| 125C>G | CYP1A2 | rs72547511 | CYP1A2*15 |
| 558C>A | CYP1A2 | rs72547513 | CYP1A2*11 |
| 2116G>A | CYP1A2 | rs56276455 | CYP1A2*3 |
| 2499A>T | CYP1A2 | rs72547516 | CYP1A2*4 |
| 3497G>A | CYP1A2 | rs55889066 | CYP1A2*5 |
| 3533G>A | CYP1A2 | rs56107638 | CYP1A2*7 |
| 5090C>T | CYP1A2 | rs28399424 | CYP1A2*6 |
| 5347C>T | CYP1A2 | rs2470890 | CYP1A2*1B |
| *1 | CYP2B6 | *1 is the reference allele and is reported by default if the other tested alleles are not detected | CYP2B6*1.001 |
| *4 | CYP2B6 | rs2279343 | CYP2B6*4 |
| *6 | CYP2B6 | contains rs2279343 and rs3745274 | CYP2B6*6.001 |
| *9 | CYP2B6 | rs3745274 | CYP2B6*9 |
| *1 | CYP2C19 | *1 is the reference allele and is reported by default if the other tested alleles are not detected | CYP2C19 *1 |
| *2 | CYP2C19 | rs4244285 | CYP2C19 *2 |
| *3 | CYP2C19 | rs4986893 | CYP2C19 *3 |
| *4 | CYP2C19 | rs28399504 | CYP2C19 *4 |
| *6 | CYP2C19 | rs72552267 | CYP2C19 *6 |
| *8 | CYP2C19 | rs41291556 | CYP2C19 *8 |
| *17 | CYP2C19 | rs12248560 | CYP2C19 *17 |
| *1 | CYP2C9 | *1 is the reference allele and is reported by default if the other tested alleles are not detected | CYP2C9*1 |
| *2 | CYP2C9 | rs1799853 | CYP2C9*2 |
| *3 | CYP2C9 | rs1057910 | CYP2C9*3 |
| *5 | CYP2C9 | rs28371686 | CYP2C9*5 |
| *6 | CYP2C9 | rs9332131 | CYP2C9*6 |
| *1 | CYP2D6 | *1 is the reference allele and is reported by default if the other tested alleles are not detected | CYP2D6 *1 |
| *2 | CYP2D6 | rs16947 | CYP2D6 *2 |
| *2A | CYP2D6 | rs1080985 | CYP2D6 *2 |
| *3 | CYP2D6 | rs35742686 | CYP2D6 *3.001 |
| *4 | CYP2D6 | rs3892097 | CYP2D6 *4 |
| *5 | CYP2D6 | CYP2D6 deletion | CYP2D6 *5.001 |
| *6 | CYP2D6 | rs5030655 | CYP2D6 *6 |
| *7 | CYP2D6 | rs5030867 | CYP2D6 *7.001 |
| *8 | CYP2D6 | rs5030865 | CYP2D6 *8 |
| *9 | CYP2D6 | rs5030656 | CYP2D6 *9.001 |
| *10 | CYP2D6 | rs1065852 | CYP2D6 *10 |
| *11 | CYP2D6 | rs5030863/rs201377835 | CYP2D6 *11 |
| *12 | CYP2D6 | rs5030862 | CYP2D6 *12 |
| *14 | CYP2D6 | rs5030865 | CYP2D6 *14 |
| *15 | CYP2D6 | rs72549357/rs774671100 | CYP2D6 *15 |
| *17 | CYP2D6 | rs28371706 | CYP2D6 *17 |
| *41 | CYP2D6 | rs28371725 | CYP2D6 *41 |
| gene duplication | CYP2D6 |  |  |
| *1 | CYP3A4 | *1 is the reference allele and is reported by default if the other tested alleles are not detected | CYP3A4 *1 |
| *13 | CYP3A4 | rs4986909 | CYP3A4 *13.001 |
| *15A | CYP3A4 | rs4986907 | CYP3A4 *15.001 |
| *22 | CYP3A4 | rs35599367 | CYP3A4 *22 |
| L/S | SLC6A4 | rs4795541 | n/a |
| -1438G>A | HTR2A | rs6311 | n/a |
| rs13429709T>C | GCG | rs13429709 | n/a |
| rs3134701A>G | HCRTR2 | rs3134701 | n/a |
| rs4142972G>A | HCRTR2 | rs4142972 | n/a |
| rs489693C>T | MC4R | rs489693 | n/a |
| rs6435326T>A | NDUFS1 | rs6435326 | n/a |
| rs16147A>G | NPY | rs16147 | n/a |

n/a: not applicable
^a^ Pharmacogene Variation Consortium: https://www.pharmvar.org/gene/

**Supplementary Table 2. Psychotropic Medications and Genes Considered in the Combinatorial Pharmacogenomic Test Panel During the GAPP-MDD Trial***

| ***Antidepressants*** |  |  |  |  |  |  |  |  |
| --- | --- | --- | --- | --- | --- | --- | --- | --- |
|  | **CYP2D6** | **CYP3A4** | **CYP1A2** | **CYP2C9** | **CYP2C19** | **CYP2B6** | **SLC6A4** | **HTR2A** |
| **Amitriptyline** | X | X | X | X | X | - | - | - |
| **Bupropion** | X | X | - | - | - | X | - | - |
| **Citalopram** | X | X | - | - | X | - | X | - |
| **Clomipramine** | X | X | X | - | X | - | - | - |
| **Desipramine** | X | - | - | - | - | - | - | - |
| **Desvenlafaxine** | - | X | - | - | X | - | - | - |
| **Doxepin** | X | X | X | X | X | - | - | - |
| **Duloxetine** | X | - | X | - | - | - | - | - |
| **Escitalopram** | X | X | - | - | X | - | X | - |
| **Fluoxetine** | X | X | - | X | X | - | X | - |
| **Fluvoxamine** | X | - | X | - | - | - | X | - |
| **Imipramine** | X | X | X | - | X | - | - | - |
| **Mirtazapine** | X | X | X | X | - | - | - | - |
| **Nortriptyline** | X | - | - | - | - | - | - | - |
| **Paroxetine** | X | X | - | - | - | - | X | X |
| **Selegiline** | - | X | X | - | X | X | - | - |
| **Sertraline** | X | X | - | X | X | X | X | - |
| **Trazodone** | X | X | X | - | - | - | - | - |
| **Venlafaxine** | X | X | - | X | X | - | - | - |
|  |  |  |  |  |  |  |  |  |
| ***Antipsychotics*** |  |  |  |  |  |  |  |  |
| **Aripiprazole** | X | X | - | - | - | - | - | - |
| **Asenapine** | X | X | X | - | - | - | - | - |
| **Chlorpromazine** | X | X | X | - | - | - | - | - |
| **Clozapine** | X | X | X | X | X | - | - | - |
| **Fluphenazine** | X | X | X | X | X | - | - | - |
| **Haloperidol** | X | X | X | - | - | - | - | - |
| **Lurasidone** | - | X | - | - | - | - | - | - |
| **Olanzapine** | X | X | X | - | - | - | - | - |
| **Paliperidone** | X | X | - | - | - | - | - | - |
| **Perphenazine** | X | X | X | - | X | - | - | - |
| **Quetiapine** | X | X | - | - | - | - | - | - |
| **Risperidone** | X | X | - | - | - | - | - | - |
| **Thiothixene** | - | - | X | - | - | - | - | - |
| **Ziprasidone** | - | X | X | - | - | - | - | - |

*The medications and genes listed in this table represent the version of the combinatorial pharmacogenomic test that was utilized in the GAPP-MDD study. Regular reviews of the current literature and internal research are performed to support test updates; this table does not reflect the current version of the combinatorial pharmacogenomic test.

**Supplementary Table 3. Comparison of HAM-D17 clinical outcomes by treatment arm between the GAPP-MDD and GUIDED clinical trials at week 8.**

| **Outcomes** | **Treatment Arm** | **GAPP-MDD Trial**  **(N=196)** | **GUIDED Trial**  **(N=1167)** |
| --- | --- | --- | --- |
| ***Per-Protocol Cohort*** | | | |
| **Symptom Improvement^a^** | **TAU, Mean % (SE)** | 22.7 (3.6) | 24.4 (1.2) |
|  | **Guided Care^d^, Mean % (SE)** | 27.6 (2.6) | 27.2 (1.3) |
|  | **Difference** | 4.9 | 2.8 |
|  | **p-value** | 0.274 | 0.107 |
| **Response^b^** | **TAU, % (SE)** | 22.7 (5.1) | 19.9 (1.6) |
|  | **Guided Care^d^, % (SE)** | 30.3 (4.1) | 26.0 (1.9) |
|  | **Difference** | 7.6 | 6.1 |
|  | **p-value** | 0.262 | 0.013 |
| **Remission^c^** | **TAU, % (SE)** | 8.3 (3.3) | 10.1 (1.2) |
|  | **Guided Care^d^, % (SE)** | 15.7 (3.4) | 15.3 (1.6) |
|  | **Difference** | 7.4 | 5.2 |
|  | **p-value** | 0.131 | 0.007 |
|  | **Treatment Arm** | **GAPP-MDD**  **(N=308)** | **GUIDED**  **(N=1298)** |
| ***Intent-to-Treat Cohort*** | | | |
| **Symptom Improvement^a^** | **TAU, Mean % (SE)** | 17.8 (3.6) | 23.5 (1.2) |
|  | **Guided Care^d^, Mean % (SE)** | 23.8 (2.4) | 26.7 (1.3) |
|  | **Difference** | 6.0 | 3.2 |
|  | **p-value** | 0.166 | 0.069 |
| **Response^b^** | **TAU, % (SE)** | 21.9 (4.2) | 19.8 (1.5) |
|  | **Guided Care^d^, % (SE)** | 25.1 (3.0) | 26.1 (1.8) |
|  | **Difference** | 3.3 | 6.3 |
|  | **p-value** | 0.538 | 0.007 |
| **Remission^c^** | **TAU, % (SE)** | 9.7 (2.9) | 11.4 (1.3) |
|  | **Guided Care^d^, % (SE)** | 16.4 (2.7) | 16.8 (1.6) |
|  | **Difference** | 6.7 | 5.4 |
|  | **p-value** | 0.103 | 0.005 |

^a^ Mean percent change in HAM-D17 score from baseline to week 8.

^b^ Rate of response in HAM-D17 at week 8.

^c^ Rate of remission in HAM-D17 at week 8.
**^d^** Guided care arms included patients in GEN and EGEN arms

**Supplementary Table 4. Demographic Characteristics at Baseline by Treatment in the Intent-To-Treat Cohort**

|  | **Treatment** | | |  |  |
| --- | --- | --- | --- | --- | --- |
|  | **TAU**  **(N=118)** | **GEN**  **(N=125)** | **E-GEN**  **(N=128)** | **Total**  **(N=371)** | **p-value^c^** |
| **Age Group, n (%)** | | | | |  |
| 18 to 34 | 44 (37.3) | 59 (47.2) | 46 (35.9) | 149 (40.2) | 0.14 |
| 35 to 49 | 38 (32.2) | 29 (23.2) | 44 (34.4) | 111 (29.9) | 0.12 |
| 50 to 64 | 29 (24.6) | 29 (23.2) | 33 (25.8) | 91 (24.5) | 0.89 |
| 65 and Over | 7 (5.9) | 8 (6.4) | 5 (3.9) | 20 (5.4) | 0.65 |
| **Age** | | | | |  |
| Mean (SD) | 41.6 (14.3) | 39.9 (15.0) | 40.7 (13.5) | 40.7 (14.2) | 0.66 |
| Min, Max | 19.0, 78.0 | 19.0, 76.0 | 18.0, 85.0 | 18.0, 85.0 | - |
| **Gender, n (%)** | | | | |  |
| Female | 75 (63.6) | 80 (64.0) | 80 (62.5) | 235 (63.3) | 0.97 |
| Male | 43 (36.4) | 45 (36.0) | 48 (37.5) | 136 (36.7) | 0.97 |
| **Ethnicity, n (%)** | | | | |  |
| Asian | 10 (8.5) | 14 (11.2) | 11 (8.6) | 35 (9.4) | 0.71 |
| Black | 1 (0.8) | 4 (3.2) | 6 (4.7) | 11 (3.0) | 0.20 |
| Caucasian | 105 (89.0) | 101 (80.8) | 104 (81.3) | 310 (83.6) | 0.16 |
| Latin American | 2 (1.7) | 2 (1.6) | 2 (1.6) | 6 (1.6) | 0.99 |
| Other | 0 | 4 (3.2) | 5 (3.9) | 9 (2.4) | 0.11 |
| **Depression Category, n (%)^a^** | | | | |  |
| None (HAM-D17 0-7) | 2 (1.7) | 1 (0.8) | 3 (2.3) | 6 (1.6) | 0.62 |
| Mild (HAM-D17 8-13) | 14 (11.9) | 15 (12.0) | 14 (10.9) | 43 (11.6) | 0.96 |
| Moderate (HAM-D17 14-18) | 33 (28.0) | 31 (24.8) | 37 (28.9) | 101 (27.2) | 0.75 |
| Severe (HAM-D17 19-22) | 25 (21.2) | 28 (22.4) | 28 (21.9) | 81 (21.8) | 0.97 |
| Very Severe (HAM-D17 > 22) | 41 (34.7) | 46 (36.8) | 42 (32.8) | 129 (34.8) | 0.80 |
| **Psychiatric Comorbidities, n (%)** | | | | |  |
| Generalized Anxiety Disorder | 51 (43.2) | 61 (48.8) | 52 (40.6) | 164 (44.2) | 0.41 |
| Panic Disorder | 11 (9.3) | 15 (12.0) | 18 (14.1) | 44 (11.9) | 0.52 |
| Post-Traumatic Stress Disorder | 11 (9.3) | 10 (8.0) | 9 (7.0) | 30 (8.1) | 0.80 |
| **HAM-D17** | | | | |  |
| Mean (SD) | 19.8 (5.6) | 20.0 (5.6) | 19.9 (6.0) | 19.9 (5.7) | 0.95 |
| Min, Max | 6.0, 36.0 | 7.0, 36.0 | 6.0, 33.0 | 6.0, 36.0 | - |
| **Number of Failed Psych Meds, n(%)** | | | | |  |
| 1, n | 37 (31.4) | 24 (19.2) | 31 (24.2) | 92 (24.8) | 0.09 |
| 2, n | 26 (22.0) | 30 (24.0) | 22 (17.2) | 78 (21.0) | 0.39 |
| 3, n | 17 (14.4) | 15 (12.0) | 22 (17.2) | 54 (14.6) | 0.50 |
| 4, n | 11 (9.3) | 21 (16.8) | 13 (10.2) | 45 (12.1) | 0.14 |
| 5, n | 9 (7.6) | 10 (8.0) | 11 (8.6) | 30 (8.1) | 0.96 |
| 6+, n | 15 (12.7) | 23 (18.4) | 27 (21.1) | 65 (17.5) | 0.21 |
| Missing, n^b^ | 3 (2.5) | 2 (1.6) | 2 (1.6) | 7 (1.9) | 0.82 |
| Mean (SD) | 2.9 (2.1) | 3.4 (2.1) | 3.7 (3.0) | 3.4 (2.4) | 0.0469 |
| Min, Max | 1.0, 9.0 | 1.0, 8.0 | 1.0, 21.0 | 1.0, 21.0 | - |

^a^ 11 patients were missing HAM-D17 scores at baseline (N=3 in TAU, N=4 in GEN, and N=4 in E-GEN).
 These patients were still included in the ITT cohort.

^b^ Patient who reported failing ≥1 prior medication trial, but did not report specific number of prior failed
 trials.

^c^ P-values were calculated based on means or percent of patients in each group

**Supplementary Table 5. Comparison of clinical outcomes in the EGEN and GEN treatment arms at week 8, according to HAM-D17 in the Per-Protocol cohort.**

| **Outcomes** | **Treatment Arm** | | **Difference** | **p-value** |
| --- | --- | --- | --- | --- |
|  | **GEN**  **(N=65)** | **EGEN**  **(N=62)** |  |  |
| **Symptom Improvement^a^, Mean % (SE)** | 24.4 (3.9) | 30.9 (4.0) | 6.4 | 0.244 |
| **Response^b^, % (SE)** | 26.4 (5.5) | 34.4 (6.1) | 8.0 | 0.332 |
| **Remission^c^, % (SE)** | 15.0 (4.7) | 16.3 (5.0) | 1.4 | 0.834 |

^a^ Mean percent change in HAM-D17 score from baseline to week 8.

^b^ Rate of response in HAM-D17 at week 8.

^c^ Rate of remission in HAM-D17 at week 8.

**Supplementary Table 6. Clinical outcomes assessed by HAM-D17 for Guided Care and Treatment As Usual over 24 weeks in the Per-Protocol cohort^†^**

| **Endpoint** | **Week** | **Treatment** | **N** | **Estimate** | **SE** | **Difference** | **p-value** |
| --- | --- | --- | --- | --- | --- | --- | --- |
| **Symptom Improvement^a^** | 4 | TAU | 73 | 18.0 | 3.8 | 1.4 | 0.759 |
|  |  | GC | 141 | 19.4 | 2.7 |  |  |
|  | 8^†^ | TAU | 69 | 22.8 | 3.9 | 4.7 | 0.326 |
|  |  | GC | 127 | 27.5 | 2.8 |  |  |
|  | 12 | TAU | 65 | 28.6 | 4.0 | 3.2 | 0.518 |
|  |  | GC | 108 | 31.8 | 3.0 |  |  |
|  | 24 | TAU | 55 | 33.7 | 4.2 | 5.5 | 0.303 |
|  |  | GC | 94 | 39.2 | 3.2 |  |  |
| **Response^b^** | 4 | TAU | 73 | 13.9 | 4.1 | -0.8 | 0.869 |
|  |  | GC | 141 | 13.1 | 2.9 |  |  |
|  | 8^†^ | TAU | 69 | 22.7 | 5.1 | 7.6 | 0.256 |
|  |  | GC | 127 | 30.3 | 4.1 |  |  |
|  | 12 | TAU | 65 | 26.9 | 5.5 | 5.2 | 0.467 |
|  |  | GC | 108 | 32.1 | 4.4 |  |  |
|  | 24 | TAU | 55 | 40.1 | 6.6 | 2.6 | 0.756 |
|  |  | GC | 94 | 42.7 | 5.1 |  |  |
| **Remission^c^** | 4 | TAU | 73 | 6.3 | 2.8 | 0.5 | 0.875 |
|  |  | GC | 141 | 6.8 | 2.2 |  |  |
|  | 8^†^ | TAU | 69 | 9.0 | 3.4 | 6.6 | 0.177 |
|  |  | GC | 127 | 15.5 | 3.4 |  |  |
|  | 12 | TAU | 65 | 13.4 | 4.2 | 6.5 | 0.269 |
|  |  | GC | 108 | 19.9 | 3.9 |  |  |
|  | 24 | TAU | 55 | 26.1 | 5.9 | 4.3 | 0.579 |
|  |  | GC | 94 | 30.4 | 4.7 |  |  |

^a^ Estimate refers to mean percent change in HAM-D17 score from baseline to week 8.

^b^ Estimate refers to rate of response (%) in HAM-D17 at week 8.

^c^ Estimate refers to rate of remission (%) in HAM-D17 at week 8.

†Data included in this table are from one model which includes all timepoints up to week 24 and therefore, the week 8 data is different from the week 8 results that were previously reported.

GC = Guided Care; TAU = Treatment As Usual

**Supplementary Table 7. Clinical outcomes assessed by HAM-D17 for Guided Care and Treatment As Usual over 24 weeks in the Intent-To-Treat cohort^†^**

| **Endpoint** | **Week** | **Treatment** | **N** | **Estimate** | **SE** | **Diff** | **p-value** |
| --- | --- | --- | --- | --- | --- | --- | --- |
| **Symptom Improvement^a^** | 4 | TAU | 101 | 11.9 | 3.7 | 4.1 | 0.358 |
|  |  | GC | 216 | 16.0 | 2.5 |  |  |
|  | 8^†^ | TAU | 97 | 17.2 | 3.8 | 6.2 | 0.175 |
|  |  | GC | 211 | 23.4 | 2.6 |  |  |
|  | 12 | TAU | 104 | 22.9 | 3.7 | 2.6 | 0.552 |
|  |  | GC | 225 | 25.5 | 2.5 |  |  |
|  | 24 | TAU | 97 | 24.4 | 3.8 | 5.5 | 0.232 |
|  |  | GC | 211 | 29.9 | 2.6 |  |  |
| **Response^b^** | 4 | TAU | 101 | 10.3 | 3.0 | 3.4 | 0.388 |
|  |  | GC | 216 | 13.8 | 2.4 |  |  |
|  | 8^†^ | TAU | 97 | 21.3 | 4.2 | 3.5 | 0.498 |
|  |  | GC | 211 | 24.9 | 3.0 |  |  |
|  | 12 | TAU | 104 | 25.2 | 4.3 | 2.5 | 0.639 |
|  |  | GC | 225 | 27.7 | 3.0 |  |  |
|  | 24 | TAU | 97 | 32.3 | 4.8 | 1.4 | 0.813 |
|  |  | GC | 211 | 33.7 | 3.3 |  |  |
| **Remission^c^** | 4 | TAU | 101 | 6.0 | 2.2 | 2.6 | 0.368 |
|  |  | GC | 216 | 8.6 | 2.0 |  |  |
|  | 8^†^ | TAU | 97 | 9.5 | 2.9 | 6.7 | 0.100 |
|  |  | GC | 211 | 16.3 | 2.7 |  |  |
|  | 12 | TAU | 104 | 15.2 | 3.5 | 4.5 | 0.315 |
|  |  | GC | 225 | 19.7 | 2.8 |  |  |
|  | 24 | TAU | 97 | 24.5 | 4.4 | 1.5 | 0.772 |
|  |  | GC | 211 | 26.0 | 3.1 |  |  |

^a^ Estimate refers to mean percent change in HAM-D17 score from baseline to week 8.

^b^ Estimate refers to rate of response in HAM-D17 at week 8.

^c^ Estimate refers to rate of remission in HAM-D17 at week 8.

†Data included in this table are from one model which includes all timepoints up to week 24 and therefore, the week 8 data is different from the week 8 results that were previously reported.

GC = Guided Care; TAU = Treatment As Usual

**Supplementary Table 8. Medication congruence from baseline to week 8 by TAU and GS arms in the Per-Protocol and Intent-To-Treat cohorts.**

| **Outcomes** | **Arm** | **Baseline** | **Week 4** | **Week 8** |
| --- | --- | --- | --- | --- |
| *Per-Protocol Cohort* | | | | |
| **% Congruent** | **TAU (N, %)** | 60/74, 81.1 | 56/69, 81.2 | 55/67, 82.1 |
|  | **Guided Care (N, %)** | 131/157, 83.4 | 123/138, 89.1 | 112/123, 91.1 |
|  | **Diff** | 2.4 | 8.0 | 9.0 |
|  | **p-value** | 0.659 | 0.114 | 0.070 |
|  | | | | |
| **Outcomes** | **Arm** | **Baseline** | **Week 4** | **Week 8** |
| *Intent-To-Treat Cohort* | | | | |
| **% Congruent** | **TAU (N, %)** | 80/97, 82.5 | 83/100, 83.0 | 86/101, 85.2 |
|  | **Guided Care (N, %)** | 177/214, 82.7 | 195/225, 86.7 | 196/217, 90.3 |
|  | **Diff** | 0.2 | 3.7 | 5.2 |
|  | **p-value** | 0.959 | 0.386 | 0.175 |

**Supplementary Table 9. Demographic Characteristics of the Per-Protocol cohort in GAPP-MDD and GUIDED trials.**

| **Demographic** | **GAPP Study**  **N = 276** | **GUIDED Study**  **N = 1398** |
| --- | --- | --- |
| **Age Group, n (%)** | | |
| 18 to 34 | 109 (39.5) | 320 (22.9) |
| 35 to 49 | 80 (29.0) | 392 (28.0) |
| 50 to 64 | 70 (25.4) | 501 (35.8) |
| 65 and Over | 17 (6.2) | 185 (13.2) |
| **Age** | | |
| Mean (SD) | 41.1 (14.1) | 47.5 (14.5) |
| **Gender** | | |
| Female, N (%) | 178 (64.5) | 987 (70.6) |
| Male, N (%) | 98 (35.5) | 411 (29.4) |
| **Ethnicity** | | |
| Asian, N (%) | 24 (8.7) | 29 (2.1) |
| Black, N (%) | 8 (2.9) | 203 (14.5) |
| Caucasian, N (%) | 232 (84.6) | 1027 (73.5) |
| Latin American, N (%) | 5 (1.8) | 111 (7.9) |
| Other, N (%) | 7 (2.5) | 28 (2.0) |
| **History of Generalized Anxiety Disorder^a^** | | |
| Yes (%) | 118 (42.8) | 212 (15.2) |
| No (%) | 158 (57.3) | 1184 (84.8) |
| **Baseline HAM-D17** | | |
| Mean (SD) | 21.4 (4.7) | 21.3 (4.2) |
| **Number of Failed Psychiatric Medications** | | |
| Mean (SD) | 3.6 (2.6) | - 1. (3.1) |

^a^ History of Generalized Anxiety Disorder was patient reported in the GAPP study and assessed using the Mini
 International Neuropsychiatric Interview (MINI) in the GUIDED study.

**Supplementary Table 10. Expanded categories for ethnicities in GAPP-MDD trial for the intent-to-treat cohort.**

| **Ethnicity by Treatment for GAPP-MDD Trial (ITT)** | | | | | |
| --- | --- | --- | --- | --- | --- |
|  | **Treatment** | | |  |  |
| **Ethnicity** | **TAU, n (%)** | **GEN, n (%)** | **E-GEN, n (%)** | **Total, n (%)** | **Category^a^** |
| Missing | 0 (0.0) | 1 (0.3) | 1 (0.3) | 2 (0.5) | Other |
| Aboriginals | 0 (0.0) | 1 (0.3) | 2 (0.5) | 3 (0.8) | Other |
| Arab | 0 (0.0) | 1 (0.3) | 0 (0.0) | 1 (0.3) | Other |
| Black | 1 (0.3) | 4 (1.1) | 6 (1.6) | 11 (3.0) | Black |
| Caucasian | 105 (28.3) | 101 (27.2) | 104 (28.0) | 310 (83.6) | Caucasian |
| Chinese | 3 (0.8) | 2 (0.5) | 2 (0.5) | 7 (1.9) | Asian |
| Filipino | 1 (0.3) | 2 (0.5) | 2 (0.5) | 5 (1.4) | Asian |
| Japanese | 0 (0.0) | 1 (0.3) | 0 (0.0) | 1 (0.3) | Asian |
| Korean | 1 (0.3) | 1 (0.3) | 0 (0.0) | 2 (0.5) | Asian |
| Latin American | 2 (0.5) | 2 (0.5) | 2 (0.5) | 6 (1.6) | Latin American |
| Other | 0 (0.0) | 1 (0.3) | 2 (0.5) | 3 (0.8) | Other |
| South Asian | 3 (0.8) | 7 (1.9) | 6 (1.6) | 16 (4.3) | Asian |
| Southeast Asian | 2 (0.5) | 0 (0.0) | 0 (0.0) | 2 (0.5) | Asian |
| West Asian | 0 (0.0) | 1 (0.3) | 1 (0.3) | 2 (0.5) | Asian |
| **Total** | **118 (31.8)** | **125 (33.7)** | **128 (34.5)** | **371 (100.0)** |  |

^a^ Refers to how each ethnicity was reported in the demographic tables.

**Supplementary Table 11. Expanded categories for ethnicities in GAPP clinical trial for the per-protocol cohort.**

| **Ethnicity by Treatment for GAPP-MDD Trial (PP)** | | | | | |
| --- | --- | --- | --- | --- | --- |
|  | **Treatment** | | |  | |
| **Ethnicity** | **TAU, n (%)** | **GEN, n (%)** | **E-GEN, n (%)** | **Total, n (%)** | **Category^a^** |
| Missing | 0 (0.0) | 1 (0.4) | 0 (0.0) | 1 (0.4) | Other |
| Aboriginals | 0 (0.0) | 1 (0.4) | 2 (0.7) | 3 (1.1) | Other |
| Arab | 0 (0.0) | 1 (0.4) | 0 (0.0) | 1 (0.4) | Other |
| Black | 1 (0.4) | 3 (1.1) | 4 (1.5) | 8 (2.9) | Black |
| Caucasian | 83 (30.1) | 72 (26.1) | 77 (27.9) | 232 (84.1) | Caucasian |
| Chinese | 0 (0.0) | 1 (0.4) | 2 (0.7) | 3 (1.1) | Asian |
| Filipino | 1 (0.4) | 1 (0.4) | 2 (0.7) | 4 (1.5) | Asian |
| Japanese | 0 (0.0) | 1 (0.4) | 0 (0.0) | 1 (0.4) | Asian |
| Korean | 1 (0.4) | 1 (0.4) | 0 (0.0) | 2 (0.7) | Asian |
| Latin American | 2 (0.7) | 2 (0.7) | 1 (0.4) | 5 (1.8) | Latin American |
| Other | 0 (0.0) | 0 (0.0) | 2 (0.7) | 2 (0.7) | Other |
| South Asian | 3 (1.1) | 6 (2.2) | 3 (1.1) | 12 (4.4) | Asian |
| Southeast Asian | 2 (0.7) | 0 (0.0) | 0 (0.0) | 2 (0.7) | Asian |
| **Total** | **93 (33.7)** | **90 (32.6)** | **93 (33.7)** | 1. **(100.0)** |  |

^a^ Refers to how each ethnicity was reported in the demographic tables.

**Supplementary Table 12. Expanded categories for race/ethnicity in GUIDED clinical trial for the per-protocol cohort.**

| **Race/Ethnicity for GUIDED Trial (PP)** | | | |
| --- | --- | --- | --- |
|  | **Frequency** | **Percent** | **Category^a^** |
| **African American, Caucasian** | 1 | 0.1 | Other |
| **American Indian or Alaskan Native** | 8 | 0.6 | Other |
| **American Indian, African American, White** | 1 | 0.1 | Other |
| **Asian** | 29 | 2.1 | Asian |
| **Asian and White** | 1 | 0.1 | Other |
| **Belizean** | 1 | 0.1 | Other |
| **Black or African American** | 203 | 14.5 | Black |
| **Black, White, Native American** | 1 | 0.1 | Other |
| **Black, White** | 2 | 0.1 | Other |
| **Cape Verdian, Italian** | 1 | 0.1 | Other |
| **Declined to answer** | 1 | 0.1 | Other |
| **Eastern European** | 1 | 0.1 | Other |
| **Indian** | 1 | 0.1 | Other |
| **Middle Eastern** | 1 | 0.1 | Other |
| **Multiple** | 2 | 0.1 | Other |
| **Multiracial** | 3 | 0.2 | Other |
| **Native Hawaiian or Other Pacific Islander** | 1 | 0.1 | Other |
| **Turkish, Chinese** | 1 | 0.1 | Other |
| **White** | 1027 | 73.5 | Caucasian |
| **White, Black- African American** | 1 | 0.1 | Other |
| **Hispanic** | 111 | 7.9 | Latin American |
| **Total** | **1398** | **100.0** |  |

^a^ Refers to how each ethnicity was reported in the demographic tables.

# **Supplementary Table 13. Phenotypes for each gene determined by the combinatorial pharmacogenomic test across treatment arms.**

|  |  | Treatment Arm | | |  |
| --- | --- | --- | --- | --- | --- |
| Gene | Phenotype^a,b^ | TAU (N=118)  N (%) | GEN (N=125)  N (%) | EGEN (N=128)  N (%) | Total (N=371)  N(%) |
| CYP1A2 | Extensive | 59 (50) | 59 (47.2) | 55 (43) | 173 (46.6) |
|  | Intermediate | 2 (1.7) | 2 (1.6) | 1 (0.8) | 5 (1.3) |
|  | Ultrarapid | 57 (48.3) | 64 (51.2) | 72 (56.2) | 193 (52) |
| CYP2B6 | Extensive | 60 (50.8) | 65 (52) | 59 (46.1) | 184 (49.6) |
|  | Intermediate | 49 (41.5) | 55 (44) | 57 (44.5) | 161 (43.4) |
|  | Poor | 8 (6.8) | 4 (3.2) | 7 (5.5) | 19 (5.1) |
|  | Ultrarapid | 1 (0.8) | 1 (0.8) | 5 (3.9) | 7 (1.9) |
| CYP2C19 | Extensive | 89 (75.4) | 92 (73.6) | 97 (75.8) | 278 (74.9) |
|  | Intermediate | 21 (17.8) | 25 (20) | 23 (18) | 69 (18.6) |
|  | Poor | 1 (0.8) | 2 (1.6) | 3 (2.3) | 6 (1.6) |
|  | Ultrarapid | 7 (5.9) | 6 (4.8) | 5 (3.9) | 18 (4.9) |
| CYP2C9 | Extensive | 63 (53.4) | 83 (66.4) | 92 (71.9) | 238 (64.2) |
|  | Intermediate | 46 (39) | 41 (32.8) | 31 (24.2) | 118 (31.8) |
|  | Poor | 9 (7.6) | 1 (0.8) | 5 (3.9) | 15 (4) |
| CYP2D6 | Extensive | 58 (49.2) | 68 (54.4) | 68 (53.1) | 194 (52.3) |
|  | Intermediate | 28 (23.7) | 31 (24.8) | 27 (21.1) | 86 (23.2) |
|  | Poor | 22 (18.6) | 19 (15.2) | 19 (14.8) | 60 (16.2) |
|  | Ultrarapid | 10 (8.5) | 7 (5.6) | 14 (10.9) | 31 (8.4) |
| CYP3A4 | Extensive | 104 (88.1) | 121 (96.8) | 120 (93.8) | 345 (93) |
|  | Intermediate | 14 (11.9) | 4 (3.2) | 7 (5.5) | 25 (6.7) |
|  | Poor | 0 (0) | 0 (0) | 1 (0.8) | 1 (0.3) |
| HTR2A^c^ | Intermediate | 62 (52.5) | 64 (51.2) | 66 (51.6) | 192 (51.8) |
|  | Normal | 23 (19.5) | 22 (17.6) | 22 (17.2) | 67 (18.1) |
|  | Reduced | 33 (28) | 39 (31.2) | 40 (31.2) | 112 (30.2) |
| SLC6A4^d^ | Intermediate | 60 (50.8) | 60 (48) | 57 (44.5) | 177 (47.7) |
|  | Normal | 34 (28.8) | 37 (29.6) | 47 (36.7) | 118 (31.8) |
|  | Reduced | 24 (20.3) | 28 (22.4) | 24 (18.8) | 76 (20.5) |

^a^ The phenotypes assigned by the combinatorial pharmacogenomic test do not always align with other organizations and labs based on differences in methods and literature evaluation.

^b^ Phenotypic designation of *alleles is covered in U.S. patent numbers 8,401,801 and 8,688,385.

^c^ Normal (homozygous for the A allele of -1438G>A) and intermediate (heterozygous for the G allele and A allele of -1438G>A) activity of HTR2A correspond with no predicted adverse drug reactions with selective serotonin reuptake inhibitors (SSRIs). Reduced (homozygous for the G allele of -1438G>A) activity has been associated with an increased risk of adverse drug reactions with certain SSRIs^2^.

^d^ Normal response (homozygous for the long promoter polymorphism) corresponds with predicted normal time to response with SSRIs. Intermediate response (heterozygous for the short and long promoter polymorphism) corresponds with predicted increased time to response with certain SSRIs. Reduced response (homozygous for the short promoter polymorphism) corresponds with predicted delayed response with SSRIs or benefit from non-selective antidepressants.

References

1. Hall-Flavin DK, Winner JG, Allen JD, et al. Using a pharmacogenomic algorithm to guide the treatment of depression. *Transl Psychiatry.* 2012;2:e172.

2. Murphy GM, Jr., Kremer C, Rodrigues HE, Schatzberg AF. Pharmacogenetics of antidepressant medication intolerance. *Am J Psychiatry.* 2003;160(10):1830-1835.
